# The Receptor Tyrosine Kinase FGFR4 Negatively Regulates NF-kappaB Signaling

**DOI:** 10.1371/journal.pone.0014412

**Published:** 2010-12-22

**Authors:** Kristine A. Drafahl, Christopher W. McAndrew, April N. Meyer, Martin Haas, Daniel J. Donoghue

**Affiliations:** 1 Department of Chemistry and Biochemistry, University of California San Diego, La Jolla, California, United States of America; 2 Moores Cancer Center, University of California San Diego, La Jolla, California, United States of America; University of Birmingham, United Kingdom

## Abstract

**Background:**

NFκB signaling is of paramount importance in the regulation of apoptosis, proliferation, and inflammatory responses during human development and homeostasis, as well as in many human cancers. Receptor Tyrosine Kinases (RTKs), including the Fibroblast Growth Factor Receptors (FGFRs) are also important in development and disease. However, a direct relationship between growth factor signaling pathways and NFκB activation has not been previously described, although FGFs have been known to antagonize TNFα-induced apoptosis.

**Methodology/Principal Findings:**

Here, we demonstrate an interaction between FGFR4 and IKKβ (Inhibitor of NFκB Kinase β subunit), an essential component in the NFκB pathway. This novel interaction was identified utilizing a yeast two-hybrid screen [1] and confirmed by coimmunoprecipitation and mass spectrometry analysis. We demonstrate tyrosine phosphorylation of IKKβ in the presence of activated FGFR4, but not kinase-dead FGFR4. Following stimulation by TNFα (Tumor Necrosis Factor α) to activate NFκB pathways, FGFR4 activation results in significant inhibition of NFκB signaling as measured by decreased nuclear NFκB localization, by reduced NFκB transcriptional activation in electophoretic mobility shift assays, and by inhibition of IKKβ kinase activity towards the substrate GST-IκBα in *in vitro* assays. FGF19 stimulation of endogenous FGFR4 in TNFα-treated DU145 prostate cancer cells also leads to a decrease in IKKβ activity, concomitant reduction in NFκB nuclear localization, and reduced apoptosis. Microarray analysis demonstrates that FGF19 + TNFα treatment of DU145 cells, in comparison with TNFα alone, favors proliferative genes while downregulating genes involved in apoptotic responses and NFκB signaling.

**Conclusions/Significance:**

These results identify a compelling link between FGFR4 signaling and the NFκB pathway, and reveal that FGFR4 activation leads to a negative effect on NFκB signaling including an inhibitory effect on proapoptotic signaling. We anticipate that this interaction between an RTK and a component of NFκB signaling will not be limited to FGFR4 alone.

## Introduction

NFκB is a transcription factor of pivotal importance as a regulator of genes that control cell differentiation, survival, and inflammatory responses in mammalian cells. Thus, NFκB has been the subject of intense research to identify clinically useful inhibitors, and to understand the intersection of NFκB signaling with signaling pathways that are important in cancer cell biology. Upon activation with TNFα, IKKβ phosphorylates IκB, the inhibitor of NFκB, which targets it for proteasomal degradation. Subsequently, NFκB is released from sequestration in the cytoplasm, permitting translocation of NFκB dimers into the nucleus where they activate the transcription of target genes [2], [3], [4], [5], [6], [7].

Members of the FGFR family of receptor tyrosine kinases are of tremendous significance in many aspects of normal development and, additionally, have been implicated in a variety of human cancers, such as FGFR4 with regards to prostate cancer [8], [9], [10]. Signaling by FGF2 has been shown to be important for inhibition of apoptosis through PI3K/AKT and IKKβ [11], [12], and FGF signaling has also been shown to decrease TNFα-induced apoptosis through activation of the p44/42 MAPK pathway [13]. Regulatory interactions between FGFR4 and NFκB signaling pathways have not previously been reported, although both pathways represent major axes of cell signaling. In this work, we describe the discovery of a two-hybrid interaction between the receptor tyrosine kinase FGFR4 and IKKβ, an important regulatory protein in the NFκB signaling pathway, and confirm this interaction in mammalian cells. We also present evidence demonstrating a negative regulatory effect upon NFκB signaling as a consequence of FGFR4 activation.

## Results

### Interaction of FGFR4 and IKKβ proteins

**Figure 1:**
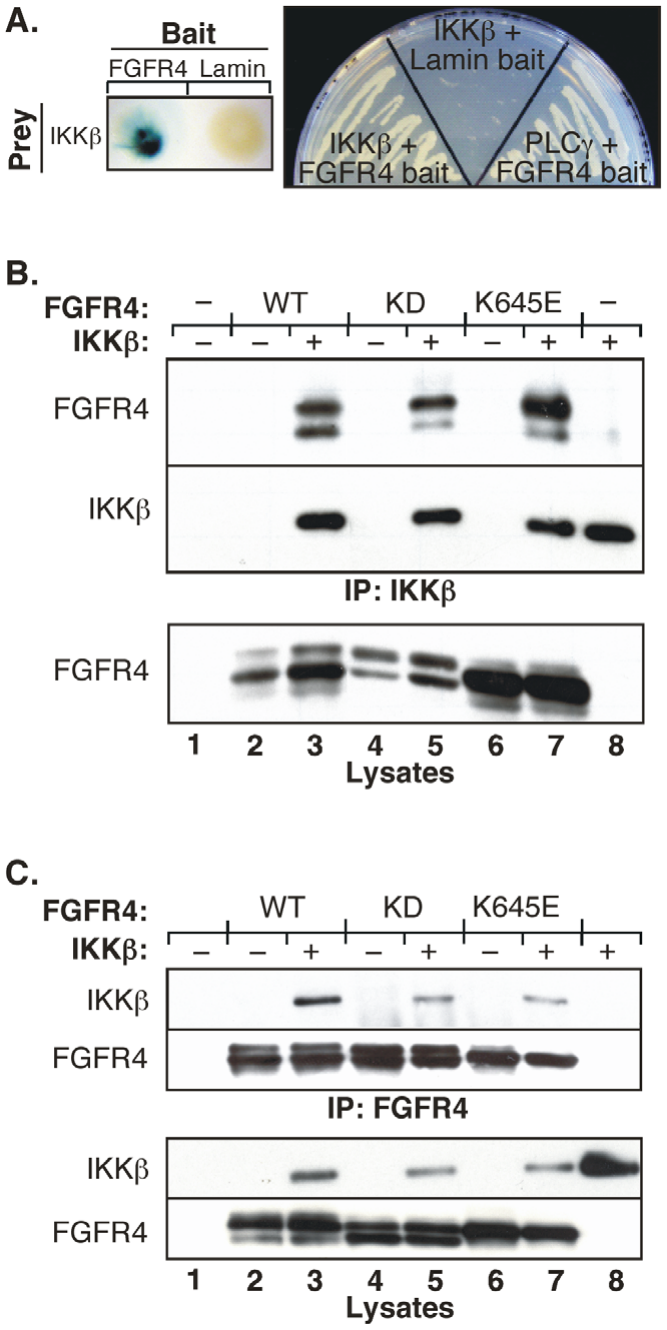
IKKβ interacts with FGFR4. **A**. Confirmation of yeast two-hybrid assay with the intracellular domain of FGFR4 bait protein and IKKβ clone isolated by β-gal filter lift assay (left panel) and growth on selective media (right panel). **B**. Full-length IKKβ and full-length FGFR4 derivatives were transfected in HEK293 cells to examine *in vivo* association. Cells were lysed in 1% NP-40 lysis buffer and immunoprecipitated with IKKβ (H-4) antibody. Immunoblot analysis was performed with FGFR4 (C-16) antibody (top panel). The membrane was stripped and reprobed with anti-IKKβ (middle panel). The expression of the FGFR4 derivatives in the whole cell lysate is shown in lower panel. **C**. Cells were transfected and lysed as in (B) then immunoprecipitated with FGFR4 (C-16) antibody. Immunoblot analysis was performed with IKKβ (H-4) antibody (top panel). The membrane was stripped and reprobed with anti-FGFR4 (second panel). The expression of IKKβ and FGFR4 in the whole cell lysate is shown in the two lower panels.

Using the intracellular domain of FGFR4 as bait, we conducted a yeast two-hybrid assay [1] and identified IKKβ as an interacting protein. The bait in this assay, fused to LexA, consisted of amino acids 373–803 of FGFR4, which includes the entirety of the intracellular domain. This was screened against a mouse embryonic cDNA library encoding fusion proteins with the VP16 transactivation domain. This novel interaction was initially detected with a β-galactosidase filter lift assay (Figure 1A, left panel), and confirmed by growth on selective media (Figure 1A, right panel). The VP16-IKKβ clone that interacted with the LexA-FGFR4 bait consisted of amino acids 607–757 of murine IKKβ (NCBI Gene: BC037723.1, NCBI Protein: NP_001153246.1), which exhibits complete identity with human IKKβ (NCBI Protein: NP_001547.1) in this region. This region includes the NEMO binding domain, residues 705–742 [14], and almost the entirety of the helix-loop-helix domain, residues 559–756, of human IKKβ [15].

To confirm the interaction of FGFR4 and IKKβ by coimmunoprecipitation using full-length proteins, human IKKβ was co-expressed with FGFR4 in HEK293 cells. IKKβ interacted with wild-type FGFR4 (FGFR4-WT), as well as with a constitutively-activated mutant of the receptor (FGFR4-K645E) (Figure 1B). Interestingly, IKKβ also interacted with a kinase-dead FGFR4 (FGFR4-KD), indicating that a functional FGFR4 kinase domain is not essential for the interaction of these two proteins. These interactions were further confirmed in the opposite direction. As before, IKKβ was detected in FGFR4 immunoprecipitates, whether kinase-active or kinase-dead (Figure 1C).

We also utilized mass spectrometry to characterize proteins recovered in IKKβ immunoprecipitates. Following expression of both the activated FGFR4-K645E and IKKβ in HEK293 cells, IKKβ immunoprecipitates were analyzed by immobilized metal affinity chromatography/nano-liquid chromatography/electrospray ionization mass spectrometry (IMAC/nano-LC/ESI-MS) [16], [17]. In two independent samples, in addition to approximately 30% coverage of IKKβ as indicated by tryptic peptides, FGFR4-derived peptides were unambiguously identified as presented in Table 1.

**Table 1:** Mass spec analysis identifies FGFR4 as binding partner of IKKβ.

Exp	Descrip	IPI Protein Index Identifier	Probability	Coverage	Peptide Sequence	Instances	Unique	AA #
1	FGFR4	IPI00304578, IPI00420109	0.9999	3%	LEIASFLPEDAGR	5	YES	86–98
1	FGFR4	IPI00304578, IPI00420109	0.9999	3%	YNYLLDVLER	6	YES	235–244
2	FGFR4	IPI00304578, IPI00420109	1	9.1%	LEIASFLPEDAGR	10	YES	86–98
2	FGFR4	IPI00304578, IPI00420109	1	9.1%	YNYLLDVLER	13	YES	235–244
2	FGFR4	IPI00304578, IPI00420109	1	9.1%	AEAFGMDPARPDQASTVAVK	2	YES	484–503
2	FGFR4	IPI00304578, IPI00420109	1	9.1%	RPPGPDLSPDGPR	1	YES	566–578
2	FGFR4	IPI00304578, IPI00420109	1	9.1%	IADFGLAR	4	NO	628–635
2	FGFR4	IPI00304578, IPI00420109	1	9.1%	NVLVTEDNVMK	6	NO	617–627
2	FGFR4	IPI00304578, IPI00420109	1	9.1%	VLLAVSEEYLDLR	2	YES	746–758

Mass spec analysis of IKKβ complexes prepared from HEK293 cells identifies FGFR4 as a binding partner. The table shows recovered FGFR4 peptides. Amino acid residues refer to the standard FGFR4 protein GenBank: AAB59389.1. Non-unique peptides appear identically within other proteins in the human proteome.

These results indicate a physical interaction between the intracellular domain of FGFR4, a receptor tyrosine kinase, and IKKβ, an important regulatory protein in NFκB signaling. The interaction described here of FGFR4 with IKKβ, or indeed with any protein involved in NFκB signaling, has not been previously reported.

### Tyrosine phosphorylation of IKKβ with FGFR4 activation

**Figure 2:**
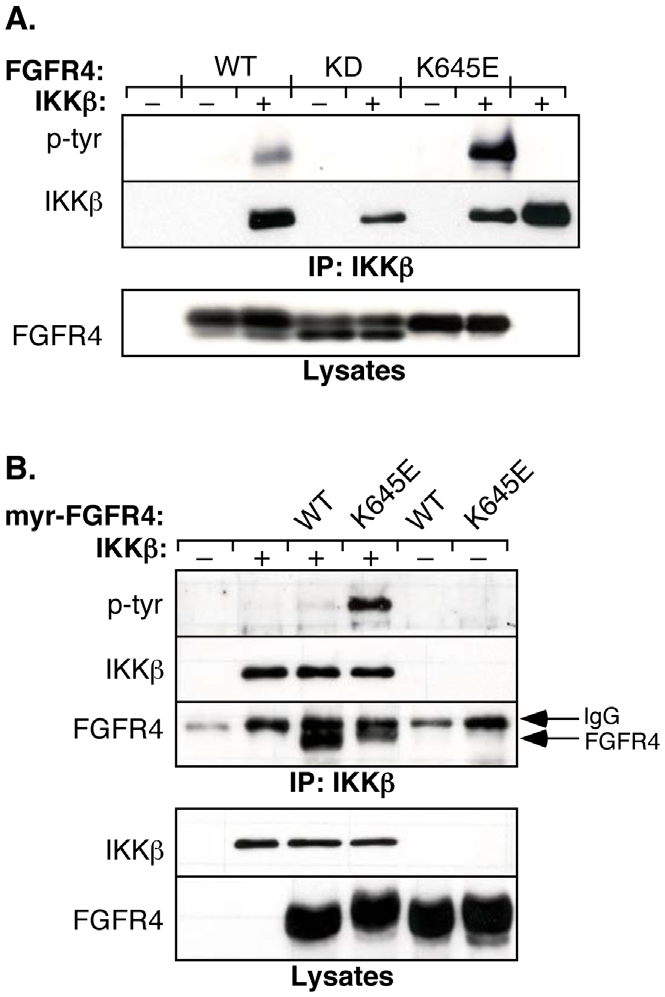
FGFR4 results in tyrosine phosphorylation of IKKβ. **A**. HEK293 cells were tranfected with IKKβ and FGFR4 derivatives. Cells were lysed in RIPA and immunoprecipitated with IKKβ (H-4) antibody. Immunoblot analysis was performed with the phosphotyrosine-specific antibody 4G10 (top panel). The membrane was stripped and reprobed with IKKβ (H-4) antibody (second panel). The expression of the FGFR4 derivatives in the lysate is shown (lower panel). **B**. HEK293 cells were tranfected with IKKβ and FGFR4 derivatives that lack their extracellular domain and are targeted to the membrane with a myristylation signal (myr-FGFR4). Cells were lysed in 1% NP-40 lysis buffer and immunoprecipitated with IKKβ (H-4) antibody. After the proteins were transferred, the membrane was cut in half and the upper part was immunoblotted with the phosphotyrosine-specific antibody 4G10 (top panel). It was stripped and reprobed with IKKβ (H-4) antibody (second panel). The lower half of the membrane was immunoblotted with FGFR4 (C-16) antibody (third panel). The lysates were examined for the expression of IKKβ and myr-FGFR4 derivatives (bottom panels).

The primary mode of IKKβ regulation is through phosphorylation of serine residues, which can be either activating as when Ser177 and Ser181 are phosphorylated, or inhibitory if phosphorylated on C-terminal residues [18], [19], [20], [21]. Tyrosine phosphorylation of IKKβ in response to growth factor receptor activation has not been previously reported. We investigated the possible tyrosine phosphorylation of IKKβ in HEK293 cells expressing FGFR4, and found that IKKβ was tyrosine phosphorylated (Figure 2). Expression of FGFR4 WT led to an increase in tyrosine phosphorylation of IKKβ, in contrast to the kinase-dead mutant of FGFR4, indicating a requirement for FGFR4 kinase activity in IKKβ tyrosine phosphorylation. Additionally, a strongly activated mutant of FGFR4 [22] led to a dramatic increase in tyrosine phosphorylation of IKKβ (Figure 2A). Importantly, all experiments were performed using a non-epitope-tagged IKKβ. In initial control experiments, we determined that the presence of the 3x-HA epitope tag (YPYDVPDYA) at the N-terminus of IKKβ resulted in a significant increase in the extent of tyrosine phosphorylation in response to FGFR4 activation (data not shown), presumably due to phosphorylation at some of the 9 Tyr residues contained within the 3x-HA-tag.

By SDS PAGE, IKKβ migrates at ∼87 kDa while the lower, unmodified band of FGFR4 almost comigrates at ∼85 kDa. To ensure that the tyrosine phosphorylation observed was on IKKβ and not autophosphorylation of FGFR4 (Figure 2A), cells were lysed in RIPA buffer, and immunoprecipitations were washed over 10% sucrose to eliminate protein-protein interactions. In addition, we examined tyrosine phosphorylation of IKKβ when cotransfected with a truncated, myristylated FGFR4 containing only the intracellular domain of FGFR4 with a myristylation signal for membrane localization [22]. Using these shorter FGFR4 constructs allowed clear separation from IKKβ, and revealed that tyrosine phosphorylation of IKKβ was still present (Figure 2B). Furthermore, we examined the interaction of these proteins and demonstrated that the myr-FGFR4 proteins still interact with IKKβ in coimmunoprecipitation experiments (Figure 2B).

These experiments thus provide an explanation as to why tyrosine phosphorylation of IKKβ may not have been previously reported, due to the presence of a Tyr-containing epitope tag on the most commonly used IKKβ vectors [21], [23], allowing artifactual Tyr phosphorylation within the epitope tag. Expression of non-tagged IKKβ in the experiments of Fig. 2, however, reveals the presence of verifiable Tyr phosphorylation within IKKβ sequences, and which is observed only in the presence of activated FGFR4 but not kinase-dead FGFR4.

### Activated and kinase-dead FGFR4 decrease TNFα-stimulated NFκB nuclear localization

**Figure 3:**
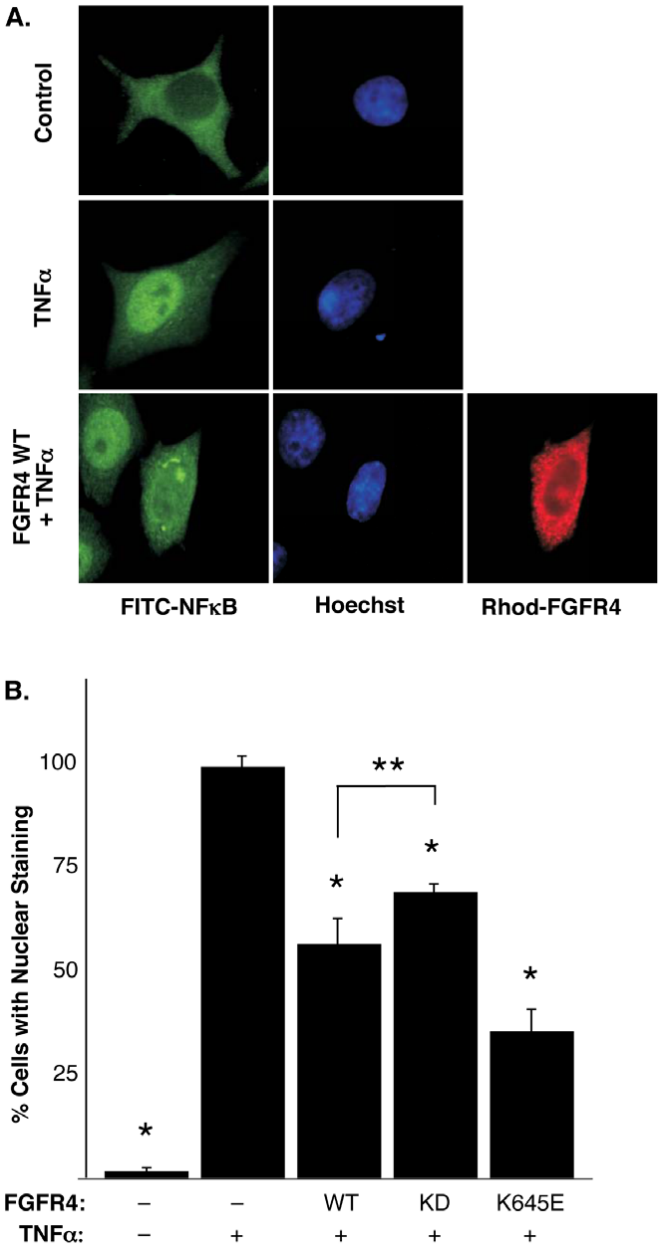
FGFR4 expression relocalizes NFκB. **A**. HeLa cells were seeded onto glass coverslips and transfected with FGFR4 derivatives. The cells were treated with TNFα for 30 min. Indirect immunofluorscence was performed. The localization of endogenous NFκB was dectected with NFκB p65 (F-6) antibody followed by FITC-conjugated anti-mouse antiserum. Cells expressing the FGFR4 derivatives were stained with anti-FGFR4 (C-16) and Rh-conjugated anti-rabbit secondary antibody. The nuclei were visualized with Hoechst dye. The endogenous localization of NFκB is shown in non-transfected cells −/+ TNFα treatment (top panels). The altered localization of NFκB in a cell expressing FGFR4 WT with TNFα treatment is shown in lower panels. **B**. Cells expressing FGFR4 derivatives were scored for the localization of NFκB. 100 cells were counted for each sample in three independent experiments. The error bars represent the standard deviation. *, P≤0.0001; **, P = 0.0061.

Utilizing indirect immunofluoresence, we monitored changes in NFκB translocation to the nucleus in TNFα stimulated cells expressing FGFR4 proteins. In starved unstimulated cells, NFκB was observed to be predominantly cytoplasmic (Fig. 3A), presumably due to sequestration by IκB as described by others [24], [25], [26], [27]. In contrast, NFκB was observed to be predominatly nuclear following TNFα stimulation. Significantly, when cells expressing FGFR4 WT were stimulated with TNFα, we observed a 40% decrease in cells exhibiting NFκB nuclear localization compared to mock-transfected cells (Fig. 3B). Expression of a constitutively-activated mutant, FGFR4-K645E, led to a 65% decrease in cells exhibiting nuclear localization of NFκB. In contrast, the kinase-dead FGFR4-KD led to only a 30% decrease in NFκB nuclear localization. These results indicate that expression of FGFR4-WT, or of the activated mutant FGFR4-K645E, results in a significant decrease in the ability of TNFα to stimulate NFκB nuclear localization. Although more modest in its effects, even FGFR-KD was able to decrease the TNFα-stimulated nuclear localization of NFκB, possibly reflecting a dominant-negative effect involving recruitment of effector molecules to a kinase-dead complex.

### FGFR4 activation decreases TNFα-stimulated IKK kinase activity assayed *in vitro*

**Figure 4:**
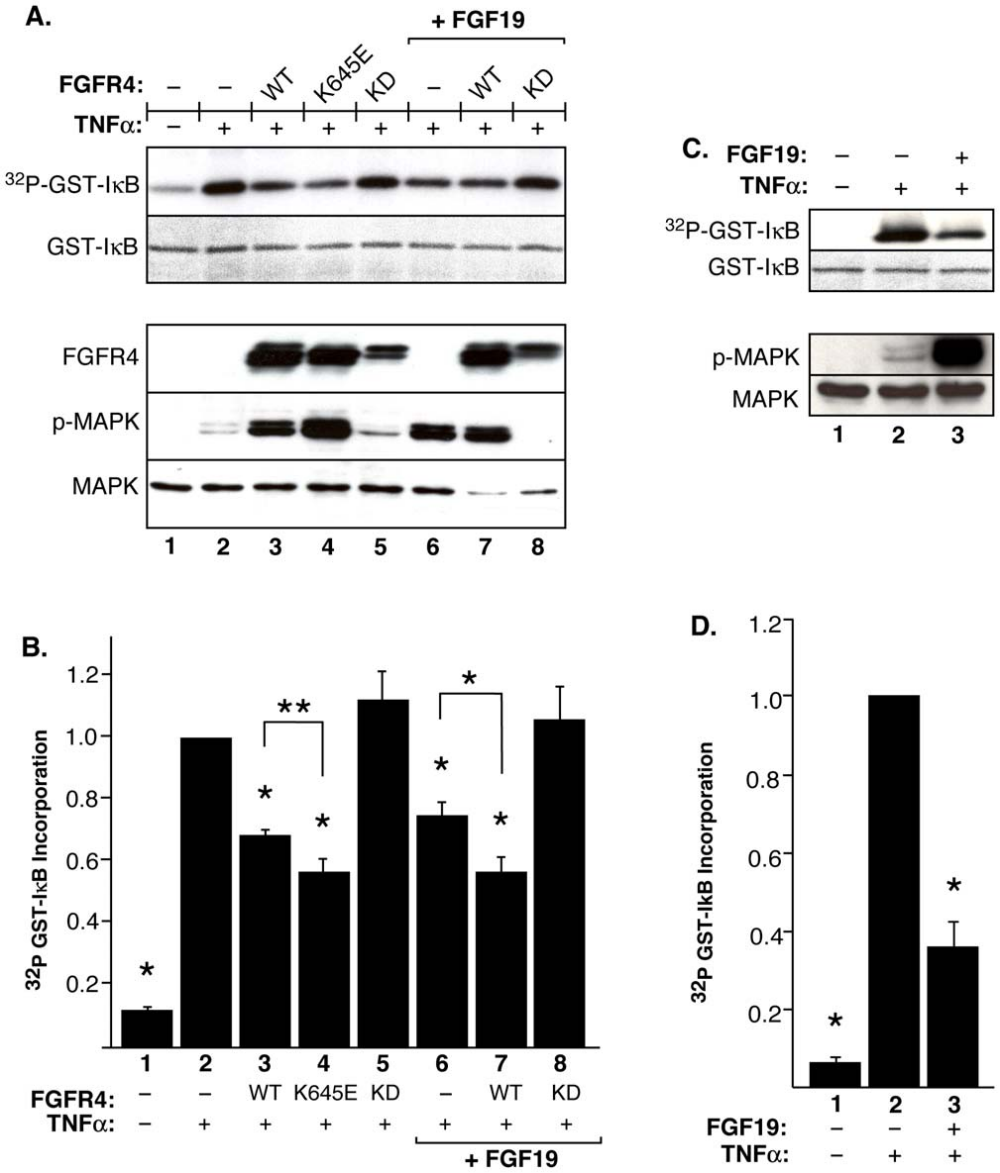
FGFR4 expression and/or FGF19 stimulation inhibits endogenous IKKβ activity. **A**. HEK293 cells were transfected with empty vector or the indicated FGFR4 constructs, then starved for 16 h. Cells were then either stimulated with vehicle for 10 min or FGF19 for 10 min prior to the addition of TNFα for an additional 10 min. The IKK complex was then immunoprecipitated from cytoplasmic extracts and subjected to an *in vitro* kinase assay utilizing GST-IκB^(1–54)^ as substrate. The top panel shows phosphorylation to produce ^32^P-GST-IκB^(1–54)^ during the *in vitro* kinase reaction, as visualized by autoradiography. The second panel shows the substrate GST-IκB^(1–54)^ present in each reaction, as determined by Coomassie staining. The lower panels show immunoblots of whole cell lysates from which immune complexes were prepared for the GST-IκB *in vitro* phosphorylation assays. These cell lysates were separated by SDS-PAGE, transferred to Immobilon-P, and probed with the indicated antibodies. **B**. Kinase reactions described in (A) were exposed to a Phosphorimager (Bio-Rad). Quantification of ^32^P incorporation into GST-IκB was performed using the Quantity One software (Bio-Rad). The average ^32^P incorporation from three independent experiments, normalized to mock-transfected cells stimulated with TNFα, is shown +/− std. dev. *, P≤0.0002; **, P = 0.004. **C**. DU145 cells were starved for 24 h prior to stimulation as described in (A). Kinase assays and immunoblots were performed as in (A). **D**. Quantification of ^32^P incorporation into GST-IκB^(1–54)^ was performed as in (B). The average ^32^P incorporation from three independent experiments, normalized to mock-transfected cells stimulated with TNFα, is shown +/− std. dev. *, P<0.0001.

To further examine the effects of FGFR4 expression on downstream NFκB signaling, changes in endogenous IKKβ activity were monitored in HEK293 cells expressing FGFR4 and/or treated with the FGFR4-specific ligand FGF19 [28]. FGFR4-WT, activated mutant FGFR4-K645E, and kinase-dead FGFR4-KD were expressed in HEK293 cells, followed by stimulation with TNFα. Immunoprecipitated IKK complexes from cell lysates were subjected to *in vitro* kinase assays utilizing GST-IκB^(1–54)^ as the substrate [24], and GST-IκB^(1–54)^ phosphorylation was visualized and quantified (Fig. 4A and B). Treatment with TNFα resulted in an almost 10-fold increase in the IKK complex activity, compared to unstimulated cells (Lane 2 versus Lane 1). Cells expressing FGFR4-WT exhibited a 30% reduction in IKK complex activity (Lane 3), which was further diminished by expression of the activated mutant FGFR4-K645E, resulting in a 45% reduction of IKK activity (Lane 4). When FGFR4-KD was examined in this assay, TNFα-stimulation of IKK complex activity was unimpaired (Lane 5). These results demonstrate that FGFR4 expression, particularly a constitutively-activated mutant, leads to significant reduction in TNFα-stimulated IKK kinase activity when assayed *in vitro*.

Importantly, when mock-transfected cells were stimulated with FGF19 to activate endogenous FGFR4 signaling (Lane 6), a significant reduction (approximately 25%) was observed in IKK complex activity. This result demonstrates that activation of the endogenous FGFR4 pathway, in the absence of overexpressed or transfected FGFR4, is sufficient to negatively regulate NFκB signaling. This negative regulation was further enhanced when cells, stimulated with TNFα+FGF19, were expressing excess FGFR4-WT (Lane 7). The inhibitory effects of FGF19 were reversed, however, when cells stimulated with TNFα+FGF19 were expressing FGFR4-KD (Lane 8). Thus, in this assay, the kinase-dead receptor exhibited a dominant-negative effect.

### Interaction of FGFR4 and NFκB pathways in DU145 prostate cancer cells

Since previous research has implicated FGFR4 in prostate cancer progression, we sought to examine the effect of FGFR4 activation on NFκB signaling in DU145 prostate cancer cells [9], [10], known to express high levels of endogenous FGFR4 [29]. When DU145 cells were stimulated with TNFα, and assayed for IKK complex activity, a significant increase was observed (Fig. 4C and D, Lane 2 versus Lane 1). When these cells were also stimulated with FGF19 in addition to TNFα, a significant decrease (approximately 65%) in IKK complex kinase activity was observed (Fig. 4C and D, Lane 3). These results demonstrate that FGF19-stimulated activation of endogenous FGFR4 in DU145 cells negatively regulates TNFα-stimulated activity of the IKK complex.

**Figure 5:**
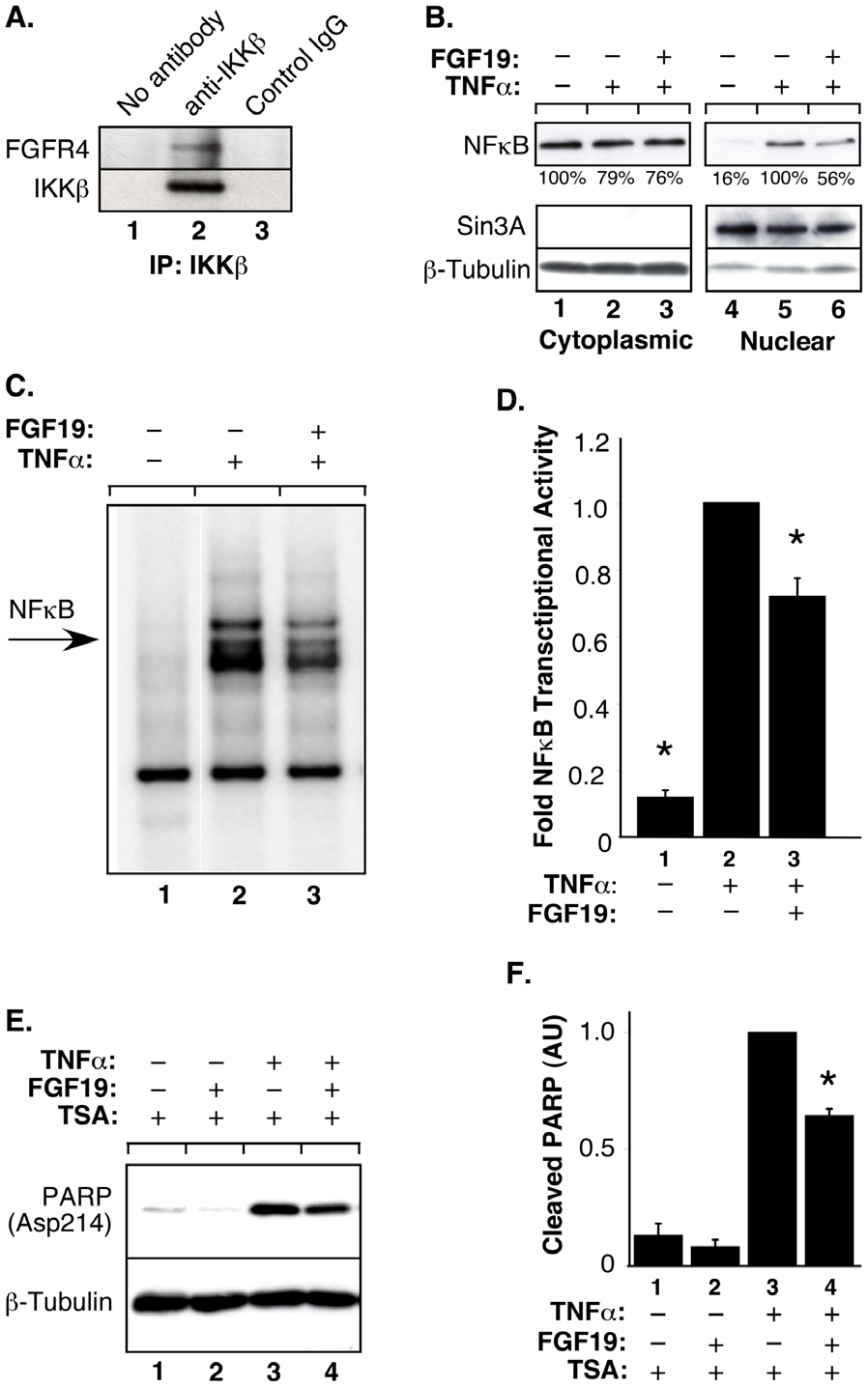
Endogenous FGFR4 and IKKβ interact in DU145 cells, and FGFR4 activation decreases TNFα-induced signaling. **A**. Approximately 500 µg of total lysate was immunoprecipitated with 2 µg IKKβ (H-4) mouse mAB in 1% NP-40 lysis buffer. Immunoblot analysis was performed with FGFR4 (C-16) antibody (top panel). The membrane was stripped and reprobed with anti-IKKβ (10AG2) (lower panel). No IKKβ (H-4) antibody was added during the immunoprecipitation for the “No antibody” control (lane 1), whereas an equal amount of normal mouse IgG was added for the “IgG” control (lane 3). **B**. DU145 cells were treated with TNFα, or TNFα + FGF19. Cells were fractionated and the cytoplasmic and nuclear fractions were immunoblotted with NFκB p65 (F-6) antibody (top panel). Membranes were stripped and reprobed with β-tubulin and mSin3A antibodies to confirm cytoplasmic and nuclear fractions (lower panels). Quantitation of cytoplasmic NFκB in Lanes 1–3, for 3 independent experiments, was normalized relative to tubulin in lower blot, with NFκB in Lane 1 set to 100%: Lane 1, 100%; Lane 2, 79%±7%; Lane 3, 76%±9%. Quantitation of nuclear NFκB in Lanes 4–6, for 3 independent experiments, was normalized relative to Sin3A in lower blot, with NFκB in Lane 5 set to 100%: Lane 4, 16%±10%; Lane 5, 100%; Lane 6, 56%±18%. **C**. DU145 cells were stimulated with vehicle for 30 min, TNFα for 30 min, or FGF19 for 10 min prior to the addition of TNFα for an additional 30 min. Nuclear extracts were prepared and equal amounts of protein (2 µg) were subjected to EMSA with ^32^P-labeled 30 bp double-stranded oligonucleotide containing a consensus κB-site. **D**. Samples from (C) were exposed to a phosphorimager (Bio-Rad). Quantification of NF-κB binding to the probe was performed using the Quantity One software (Bio-Rad). The average NF-κB binding from three independent experiments, normalized to mock-transfected cells stimulated with TNFα, is shown +/− std. dev. *, P<0.0001. **E**. DU145 cells were treated with TSA, FGF19 and TNFα as indicated. Cell lysates were separated by SDS-PAGE and transferred to Immobilon-P. The membrane was cut and the top was incubated with antibody against cleaved-PARP (top panel). The lower portion was probed with β-tubulin antibody (bottom panel). **F**. The experiment from (E) was performed in triplicate and quantitated, with the amount of cleaved PARP normalized to the TNF sample, shown +/− sem. *, P<0.03.

We also examined the interaction of endogenous IKKβ and FGFR4 in DU145 cells. As shown in Fig. 5A (Lane 2), this experiment revealed that endogenous FGFR4 protein can be recovered in an IKKβ immune complex. In addition, we examined NFκB localization in DU145 cells following treatment with TNFα and/or FGF19. Although we previously used indirect immunofluoresence, we found that DU145 cells did not sit down well on coverslips and produced equivocal images. Thus, we used cell fractionation to prepare nuclear and cytoplasmic fractions from DU145 cells. While NFκB was primarily cytoplasmic in untreated cells (Fig. 5B, compare Lanes 1 and 4), TNFα stimulation resulted in significant nuclear localization of NFκB (Lane 5). When DU145 cells were stimulated with TNFα, and also treated with FGF19, the nuclear localization of NFκB was significantly reduced to a level of 56% relative to TNFα alone (Lane 6, compare with Lane 5 which was set arbitrarily to 100%). Lastly, we examined the effects of FGF19 treatment on TNFα-induced NFκB DNA binding using EMSA assays (Figs. 5C and D). Compared with unstimulated DU145 cells, TNFα stimulated significant NFκB binding activity (Lane 2, compare with Lane 1). The addition of FGF19 decreased NFκB DNA binding activity by about 25% as measured by EMSA (Lane 3).

Using multiple assays, these experiments thus demonstrate that stimulation of the endogenous FGFR4 receptor in DU145 cells exerts an unequivocal negative regulatory effect on TNFα-stimulated outcomes.

### FGF19 stimulation reduces TNFα-induced apoptosis in DU145 cells

Next we examined the effect of FGF19 treatment on TNFα-induced apoptosis in the DU145 prostate cancer cell line. Since this cell line has previously been found to be resistant to apoptosis induced by TNF-family ligands, we utilized trichostatin A (TSA), a histone deacetylase inhibitor, to sensitize the cells to TNFα [30], [31]. DU145 cells were treated with TSA and FGF19 prior to the addition of TNFα. Cells were examined for Poly(ADP-ribose) Polymerase (PARP) cleavage as an indicator of apoptosis. FGF19 treatment reduced the amount of cleaved PARP induced by TNFα by approximately 35% (Figs. 5E and F). These results indicate that activation of FGFR4 signaling pathways in DU145 cells by FGF19 is able to negatively regulate apoptosis induced by TNFα stimulation.

### FGF19 treatment alters global TNFα-induced gene expression in DU145 cells

**Figure 6:**
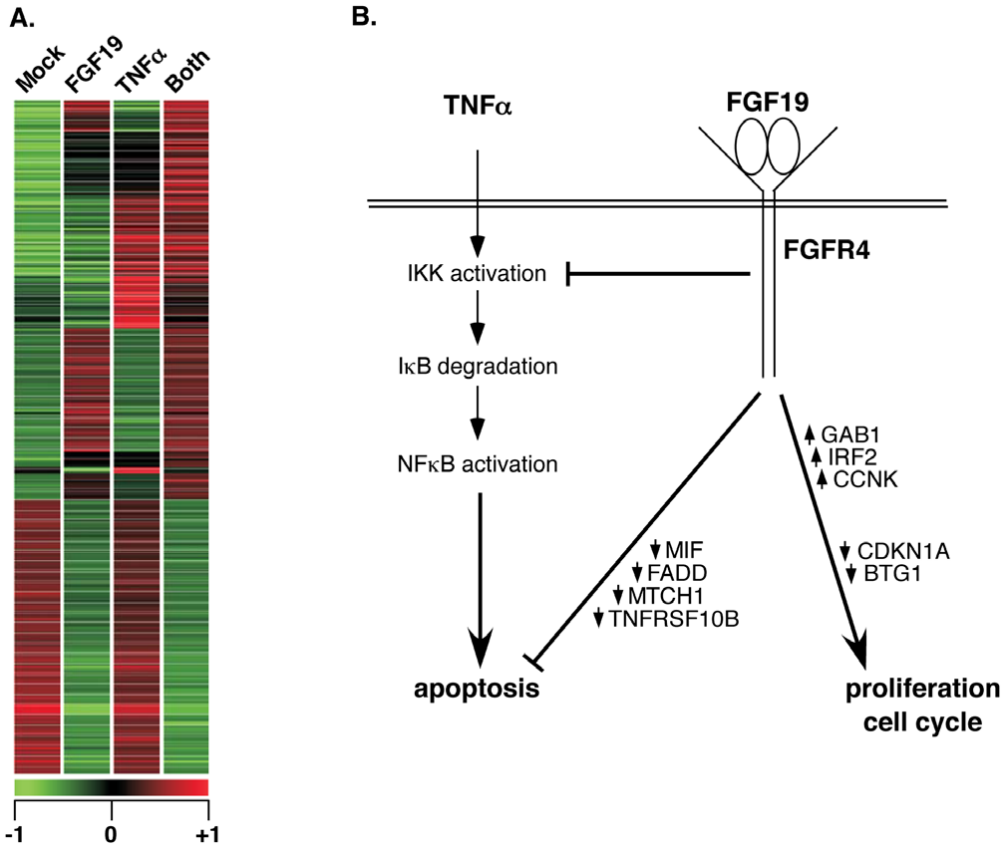
FGF19 alters TNFα-stimulated Gene Expression. **A**. Microarray analysis profiles global expression changes in DU145 cells at 1.5 h after stimulation −/+ FGF19 and −/+ TNFα. Heat map presents expression changes for probesets at a p-value cut-off of 0.015, and which satisfy a fold change cut-off of 2.0 relative to the [Mock] sample. Complete data are presented in Supporting Information Table S1. **B**. Schematic showing possible interactions between FGFR4 and NFκB pathways.

Changes in global gene expression were quantified by microarray analysis using DU145 cells treated with TNFα, FGF19, or both, and harvested at 1.5 h. Using Mock (−FGF19/−TNFα) as the control condition, 1148 out of 24,220 probesets satisfied a corrected p-value cut-off of 0.015 using ANOVA analysis; furthermore, of these, 307 satisfied a fold-change cut-off of 2.0. These results are presented graphically in the heat map shown in Fig. 6A, revealing that significant changes in global gene expression occur in DU145 cells treated with or without FGF19, and with or without TNFα, as early as 1.5 h. See Supporting Information Table S1 for complete data.

The microarray expression data were reanalyzed using the same statistical cutoff as before, but with the [+TNFα] as the control condition. Approximately 260 probesets exhibited a fold change cut-off of 2.0 or more (Supporting Information Table S2). A subset of these is presented in Table 2, showing all genes involved in the regulation of cell cycle, apoptosis, or NFκB signaling. The stimulation of DU145 cells with FGF19 + TNFα, in comparison to TNFα alone, exhibits the following general effects: 1) stimulation of cell proliferation by upregulation of proliferative genes such as GAB1, IRF2, and CCNK; 2) stimulation of cell proliferation by downregulation of cell cycle inhibitory genes such as CDKN1A and BTG1; 3) inhibition of genes involved in regulation of NFκB signaling, such as TNFRSF10B and FADD, and 4) inhibition of apoptotic responses by downregulation of genes such as MIF and MTCH1.

**Table 2:** Effects of FGF19 + TNFα treatment vsTNFα alone on gene expression in DU145 Cells.

Protein/Gene	Regulation[FGF+TNF] vs [TNF]	Fold change[FGF+TNF] vs [TNF])	RefSeq	Relevant Gene Ontology Biological or Molecular Designations
**LRRCC1** // leucine rich repeat and coiled-coil domain 1	UP	2.26	NM_033402	Cell cycle.
**GAB1** // GRB2-associated binding protein 1	UP	2.07	NM_207123	Cell proliferation.
**IRF2** // interferon regulatory factor 2	UP	2.03	NM_002199	Cell proliferation.
**CCNK** // cyclin K	UP	2.01	NM_001099402	Cell cycle.
**DDIT4** // DNA-damage-inducible transcript 4	DOWN	3.22	NM_019058	Apoptosis.
**MIF** // macrophage migration inhibitory factor	DOWN	2.42	NM_002415	Inflammatory response.
**TNFRSF10B** // tumor necrosis factor receptor superfamily, member 10b	DOWN	2.37	NM_003842	Activation of caspase activity;Activation of NF-kappaB-inducing kinase activity;Induction of apoptosis via death domain receptors; Activation of pro-apoptotic gene products.
**FADD** // Fas (TNFRSF6)-associated via death domain	DOWN	2.35	NM_003824	Induction of apoptosis via death domain receptors; Activation of pro-apoptotic gene products;Regulation of apoptosis;Positive regulation of I-kappaB kinase/NF-kappaB cascade.
**SLC35B2** // solute carrier family 35, member B2	DOWN	2.33	NM_178148	Positive regulation of I-kappaB kinase/NF-kappaB cascade.
**MTCH1** // mitochondrial carrier homolog 1	DOWN	2.32	NM_014341	Activation of caspase activity;Positive regulation of apoptosis.
**UBB** // ubiquitin B	DOWN	2.26	NM_018955	Cell cycle.
**BRMS1** // breast cancer metastasis suppressor 1	DOWN	2.22	NM_015399	Negative regulation of cell cycle.
**BTG1** // B-cell translocation gene 1, anti-proliferative	DOWN	2.16	NM_001731	Negative regulation of cell proliferation;Regulation of apoptosis.
**DUSP1** // dual specificity phosphatase 1	DOWN	2.13	NM_004417	Cell cycle.
**PLEKHG4** // pleckstrin homology domain containing, family G (with RhoGef domain) member 4	DOWN	2.11	NM_015432	Cell death.
**FNTB** // farnesyltransferase, CAAX box, beta	DOWN	2.09	NM_002028	Negative regulation of cell proliferation.
**TNIP2** // TNFAIP3 interacting protein 2	DOWN	2.06	NM_024309	I-kappaB kinase/NF-kappaB cascade.
**CDKN1A** // cyclin-dependent kinase inhibitor 1A (p21Cip1)	DOWN	2.04	NM_078467	Cell cycle arrest; Negative regulation of cell proliferation.
**CHTF8** // CTF8, chromosome transmission fidelity factor 8	DOWN	2.03	NM_001039690	Cell cycle.
**CHMP1A** // chromatin modifying protein 1A	DOWN	2.02	NM_001083314	Cell cycle.
**CKS2** // CDC28 protein kinase regulatory subunit 2	DOWN	2.00	NM_001827	Cell cycle; Cell proliferation.

Microarray expression data are presented for all genes involved in the regulation of cell cycle, apoptosis, or NFκB signaling, and which satisfy a fold-change cut-off of 2.0 and p-value cut-off of 0.015. For complete details and information, see Supporting Information Table S2.

## Discussion

In this report, we characterize a novel interaction between a receptor tyrosine kinase, FGFR4, and a key regulatory protein in the NFκB pathway, IKKβ. This interaction was initially identified by yeast two-hybrid screening (Fig. 1A), confirmed by coimmunoprecipitation in both directions in HEK293 cells (Fig. 1B and 1C), and subsequently validated by the identification of FGFR4-derived peptides by mass spectrometry analysis of IKKβ immune complexes (Table 1). Furthermore, we demonstrate that endogenous FGFR4 and IKKβ proteins interact in the DU145 prostate cancer cell line (Fig. 5A). This latter result is significant, as otherwise one could argue that the protein-protein interaction results from overexpression in HEK293 cells. We have additionally demonstrated a similar protein-protein interaction between the related receptor FGFR2 and IKKβ (data not shown). Although it seems likely that this may represent a direct interaction between these two proteins, at present, we cannot exclude the possibility that an additional unidentified protein may be involved in mediating this interaction.

These results raise the question of the biological significance of this interaction. In one approach to this question, we examined the kinase activity of IKKβ complexes recovered from cells expressing different mutants of FGFR4, using phosphorylation of GST-IκB^(1–54)^ as the readout. We show that expression of FGFR4-WT or an activated FGFR4 K645E mutant, but not kinase-dead FGFR4, leads to a decrease in the *in vitro* kinase activity of endogenous IKKβ complexes (Fig. 4A and 4B), indicating that FGFR4 kinase activity is required for the reduction in IKKβ activity. Moreover, stimulation of endogenous FGFR4 with the ligand FGF19 leads to a decrease in the kinase activity of IKKβ complexes prepared from either HEK293 or DU145 cell lines (Fig. 4). In an alternate approach, we show that expression of FGFR4 and/or stimulation of endogenous FGFR4 with FGF19 leads to a reduction in NFκB nuclear localization as revealed by immunofluorescence localization (Fig. 3) and by cell fractionation (Fig. 5B). In a third approach, we also demonstrate a decrease in the amount of NFκB DNA binding using EMSA assays (Fig. 5C and 5D). In the three different cell lines used, similar effects of FGF19/FGFR4 activation were observed with regards to the downregulation of NFκB signaling. From these assays, we conclude that FGFR4 activation overall exerts an inhibitory effect upon IKKβ activity and NFκB signaling.

Using DU145 prostate cancer cells, we demonstrate that FGF19 stimulation results in a decrease in TNFα-induced apoptosis (Fig. 5E and 5F). In addition, we utilized microarray expression analysis to profile global changes in gene expression in a short time interval (1.5 h) following treatment of DU145 cells with FGF19, TNFα, or both. When microarray data for DU145 cells stimulated with FGF19 + TNFα were compared with cells stimulated with TNFα alone, we found that the addition of FGF19 in general favored proliferative changes, while decreasing the expression of inflammatory and apoptotic genes (Table 2). Key examples of proliferative functionalities are: the increased expression of GAB1 (GRB2-associated binding protein 1), which stimulates Ras/MAPK activity [32]; the increased expression of CCNK, cyclin K, which activates CDK9 and downregulates p27^Kip1^ [33], [34]; the downregulation of CDKN1A, the cyclin-dependent kinase inhibitor p21^Cip1^ [35]; and the downregulation of BTG1, a member of an anti-proliferative gene family that regulates cell growth and differentiation [36]. On the other hand, prominent examples of anti-apoptotic changes are: the decreased expression of the proinflammatory mediator MIF (macrophage migration inhibitory factor) [37]; decreased expression of TNFRSF10B (TNF receptor superfamily, member 10b), also known as TRAIL-R2 or DR5, a Death Receptor directly involved in apoptosis [38]; decreased expression of FADD (FAS-associated death domain protein), which functions as an adapter protein in assembly of the death-inducing signaling complex [39]; and decreased expression of the pro-apoptotic mitochondrial outer membrane protein MTCH1 (mitochondrial carrier homolog 1), also known as Presenilin 1-associated protein [40]. We interpret these changes to be generally pro-proliferative and anti-apoptotic in nature, without over-interpreting the importance of altered expression of any individual gene, which would require further detailed analysis.

The data presented in Fig. 2 demonstrate tyrosine phosphorylation of IKKβ in cells expressing a kinase-active FGFR4, but not kinase-dead FGFR4. The simplest interpretation of this result would be that FGFR4 directly phosphorylates IKKβ and modulates its activity and/or stability. However, many other proteins are likely to be recruited into a complex with FGFR4 and IKKβ, and so the possibility exists that IKKβ tyrosine phosphorylation may be the result of an ancillary protein kinase in the complex. Other FGFR family members have been shown to recruit a variety of regulatory proteins including Grb2-SOS [41], Pyk2/RAFTK [42], RSK2 [43], SH2-B [44] and others; any of these might mediate effects through interaction with NFκB family members. Although beyond the scope of the present paper, using mass spectrometry, we have identified multiple sites of Tyr phosphorylation on IKKβ (data not shown). Understanding the role of these multiple phosphorylation sites is an ongoing area of research and will require significant effort to unravel. We have also demonstrated that coexpression of IKKβ with other members of the FGFR family, FGFR1, FGFR2, and FGFR3, results in IKKβ Tyr phosphorylation (data not shown); thus we are confident that the interaction we report here is not restricted to FGFR4 alone.

Several previous studies have reported activation of NFκB signaling downstream of RTKs. For example, EGF stimulation of EGFR in A431 cells or in mouse embryo fibroblasts enhanced the degradation of IκBα and resulted in NFκB activation [45]. Using non-small cell lung adenocarcinoma cell lines, this effect was subsequently shown to require phosphorylation of IκBα Tyr-42 and to be independent of IKK [46]. EGF treatment of ER-negative breast cancer cells also led to NFκB activation and indirectly, through increased expression of cyclin D, increased cell cycle progression [47]. Overexpression of the related receptor, ErbB2, in MCF-7 breast carcinoma cells resulted in enhanced NFκB activation in response to ionizing radiation [48]. A recent study [49] analyzing a prostate cancer tissue microarray documented a significant role of ErbB/PI3K/Akt/NFκB signaling in the progression of prostate cancer. These studies thus present a fairly consistent picture of NFκB activation downstream of EGFR activation.

In contrast, however, inhibition of EGFR in cervical carcinoma cells by the small molecule inhibitor PD153035 led to a dose-dependent increase in NFκB activation [50]. In studies of an unrelated RTK, activation of Ron by its ligand, hepatocyte growth factor-like protein, decreases TNFα production in alveolar macrophages after LPS challenge, resulting in decreased NFκB activation and increased IκB activity [51]. Thus, it seems clear that the interplay between the many different human RTKs with NFκB signaling components will be complex and most likely will depend on cell type and specific conditions.

FGFR4 is widely expressed during development, especially during myogenesis and development of endodermally derived organs [52], [53]. In addition, FGFR4 may be constitutively-activated or overexpressed in a variety of human neoplasias, including hepatocellular carcinoma [54], [55], prostate cancer [9], [56], rhabdomyosarcoma [57] and breast cancer [58], [59], and the potential utility of FGF19 and/or FGFR4 as a target for growth inhibition has been proposed [54], [60], [61]. While chronic FGFR stimulation can undoubtedly serve as a driver for cellular proliferation, the results reported here indicate a more complex relationship in that FGFR4 also clearly interacts with IKKβ. FGFR4 activation leads to an inhibitory effect on NFκB signaling, including an inhibitory effect on proapoptotic signaling mediated by NFκB pathways.

## Materials and Methods

### Cell culture

HeLa and HEK293 cells were grown in DMEM with 10% FBS and 1% Pen/strep; DU 145 cells were grown in RPMI1640 with 10% FBS and 1% Pen/strep. HeLa and DU145 cells were maintained in 5% CO_2_; HEK-293 cells were maintained in 10% CO_2_. Cell lines were obtained from ATCC (American Type Culture Collection) (http://www.atcc.org/).

### Plasmid constructs

The full-length FGFR4-WT and constitutively active FGFR4-K645E were described previously [22]. The kinase dead (K504M) and E681K derivatives were generated by QuikChange site-directed mutagenesis (Stratagene). The HA-IKKβ clone was received from Dr. Mark Hannink (University of Missouri). The HA-tag was removed by QuikChange site-directed mutagenesis and confirmed by DNA sequencing. The GST-IκB^(1–54)^ plasmid was provided by Prof. Alexander Hoffmann (UCSD).

### Antibodies, reagents, immunoprecipitation and immunoblot

Antibodies were obtained from the following sources: FGFR4 (C-16), IKKβ (H-4), IKKβ (10AG2), NFκB p65 (F-6), β-tubulin (H-235), IKKγ (FL-419), normal mouse IgG (sc-2025) from Santa Cruz Biotechnology; phospho-p44/42 MAPK (Thr202/Tyr204; E-10) and cleaved PARP (Asp214) from Cell Signaling; MAPK (ERK1+ERK2) from Zymed; 4G10 (antiphosphotyrosine) from Upstate Biotechnology; horseradish peroxidase (HRP) anti-mouse, HRP anti-rabbit from GE Healthcare; fluorescein-conjugated anti-mouse from Sigma and rhodamine-conjugated anti-rabbit from Boehringer-Mannheim. FGF19 and TNFα were obtained from R&D. mSin3A antibody (Santa Cruz, K-20) was a gift from Dr. Alexander Hoffmann. Poly(Glu, Tyr) was obtained from Sigma. Trichostatin A (TSA) was a gift from Dr. Leor Weinberger (UCSD). Techniques for immunoprecipitation and immunoblotting were as described previously [22], [42], [62]. Endogenous protein interactions were detected by coimmunoprecipitation using 500 µg of total cell lysate as previously described [42], [44]. To examine the effect of FGF19 stimulation on TNFα-induced apoptosis, DU145 cells were starved overnight, pre-treated with 100 ng/ml TSA as previously described [30], followed by 50 ng/ml FGF19 plus 50 µg/ml heparin for 25 min, after which TNFα was added at 1 ng/ml for 3 h.

### Yeast two-hybrid assay

The yeast two-hybrid assay was conducted as described [1], [63]. Briefly, the *Saccharomyces cerevisiae* strain L40 generated by Dr. Stan Hollenberg was transformed with derivatives of pBTM116 (constructed by Dr. Paul Bartel and Dr. Stan Fields). A LexA bait plasmid was constructed containing the juxtamembrane and intracellular region of FGFR4 (amino acids 373–803), fused in frame with LexA in pBTM116. This was screened against a 9.5 d.p.c. mouse embryonic cDNA library encoding fusion proteins with the transactivation domain of pVP16, kindly provided by Dr. Stan Hollenberg. Controls for two-hybrid assays, LexA-lamin as a negative control, and VP16-PLCγ as a positive control, were previously described [63]. The two-hybrid screen, His± minimal media assays, lacZ reporter β-galactosidase filter assay, and the use of controls were performed as previously described [63].

### Indirect immunofluorescence

Techniques for indirect immunofluorescence have been previously described [22], [42], [62]. Briefly, HeLa cells plated on glass coverslips were transfected using Fugene 6 (Roche) or calcium phosphate precipitation, starved the following day for 24 h, and treated with TNFα for 30 min prior to fixation.

### *In vitro* kinase assays

HEK293 or DU145 cells were transfected as indicated prior to overnight starvation in DMEM, then treated with 25 ng/ml FGF19 for 10 min and/or followed by 10 ng/ml TNFα for 10 min. Cells lysates were prepared, immunoprecipitated with IKKγ antibody, collected on Protein A-Sepharose beads, and subjected to *in vitro* kinase assay utilizing GST-IκB^(1–54)^ as the substrate [24], [64], [65]. *In vitro* kinase assays containing 1 µCi [γ-^32^P]-ATP in a total of 20 µM ATP were incubated at 30°C for 30 min, separated by 10% SDS-PAGE, exposed to film or phosphorimager screen, and quantitated.

### Electrophoretic Mobility Shift Assay (EMSA)

EMSA assays were as described elsewhere [66]. Briefly, 2 µg of total nuclear protein was reacted at room temperature for 15 min with excess ^32^P-labeled 30 bp double-stranded oligonucleotide (AGCTTGCTACAA**GGGACTTTCC**GCTGTCTACTTT) containing a consensus κB-site in 6 µl binding buffer (10 mM Tris-HCl pH 7.5, 50 mM NaCl, 10% glycerol, 1% NP-40, 1 mM EDTA, 0.1 µg/µl Poly(dI,dC)). Complexes were resolved on a non-denaturing 5% acrylamide gel containing 5% glycerol, and visualized and quantified using a Phosphorimager (Bio-Rad). Experimental details and probe specificity have been described [67].

### NFκB localization by cell fractionation

DU145 cells were plated on 10 cm dishes. Upon reaching 80% confluency, cells were starved overnight and treated the next day with 50 ng/ml FGF19 and 1 µg/ml heparin for 10 min prior to the addition of 10 ng/ml TNFα for 30 min. Cell lysates were fractionated as for EMSA.

### Mass spectrometry analysis

HEK293 cells were plated (3×10^6^ per 15 cm dish, 10 dishes total), 1 day prior to transfection with expression plasmids for both the activated FGFR4-K645E and IKKβ. After an additional 24 h, cell lysates were prepared as described [16], [17]. IKKβ immune complexes were prepared by incubation with IKKβ (H-4) antiserum at 4°C overnight, collected with Protein A-sepharose for an additional 2 h, and then trypsinized in 2 M urea. Peptides were analyzed by the Proteomics Facility of the Sanford-Burnham Medical Research Institute using immobilized metal affinity chromatography/nano-liquid chromatography/electrospray ionization mass spectrometry (IMAC/nano-LC/ESI-MS) [16], [17].

### Microarray expression analysis

DU145 cells were plated (8×10^5^ per 10 cm dish), and the following day cells were starved for 24 h. Cells were treated with 50 ng/ml FGF19 and 50 µg/ml heparin for 10 min prior to the addition of 10 ng/ml TNFα for 1.5 h. RNA was isolated using RNA-BEE (Tel-Test) per manufacturer's protocol. RNA was analyzed by the UCSD Moores Cancer Center Microarray Shared Resource using Affymetrix GeneChip Human Gene 1.0 ST Arrays (# 901085). Duplicate samples were analyzed in duplicate microarrays, and data were further analyzed by VAMPIRE and GeneSpring. All data is MIAME compliant and the raw data have been deposited in the Gene Expression Omnibus (GEO) (accession number GSE22807).

## Supporting Information

Table S1 Microarray Expression Data of DU145 Cells.
(0.03 MB PDF)

Table S2 Microarray Expression Data of DU145 Cells Using TNFalpha as Control.
(0.12 MB PDF)
