# Supplementary material for: The Receptor Tyrosine Kinase FGFR4 Negatively Regulates NF-kappaB Signaling
Source: PLoS One. 2010 Dec 22;5(12):e14412. doi: 10.1371/journal.pone.0014412 (PMC3008709; doi:10.1371/journal.pone.0014412)
Supplement: Table S1 — Microarray Expression Data of DU145 Cells. (0.03 MB PDF) [file pone.0014412.s001.pdf]

Supplemental Table 1. Microarray Expression Data of DU145 Cells.

Samples: DU145 cells collected 1.5 h after treatments: **1)** Mock; **2)** FGF19; **3)** TNFalpha; **4)** FGF19 + TNFalpha. Duplicate samples collected; analyzed on 8 chips; grouped in 4 treatments for analysis.

Analysis: Analysis in GeneSpringGX: 1148 out of 24,220 probesets satisfy corrected p-value cut-off of 0.015 using Oneway ANOVA Asymtotic p-value computation. Of these, 307 shown here satisfy fold-change cut-off of 2.0 in at least one condition pair, using Sample 1 [Mock] as the control condition.

Transcript Cluster ID refers to Affymetrix GeneChip Human Gene 1.0 ST Array Human (cat# 901085).
Information on Probesets is available at http://media.affymetrix.com in the file HuGene-1_0-v1.na30.hg19.transcript.csv.zip

Transcript Cluster ID	Fold change [FGF] vs [Mock]	Regulation [FGF] vs [Mock]	Fold change [TNF] vs [Mock]	Regulation [TNF] vs [Mock]	Fold change [Both] vs [Mock]	Regulation [Both] vs [Mock]
7899016	2.2608542	down	1.0809926	down	2.4445314	down
7899455	2.4446414	up	1.0935985	up	2.7450182	up
7900922	2.4944987	down	1.0960414	down	2.3818767	down
7901054	1.388867	down	2.6486068	up	1.3617798	up
7901110	2.5400648	down	1.0070043	down	2.459696	down
7901175	2.0707376	down	1.1713148	down	2.3878517	down
7901993	1.8517549	up	1.5409014	up	2.5022717	up
7903786	1.1968074	up	2.0255053	up	1.981951	up
7905085	2.517727	down	1.0637678	up	2.3968148	down
7905881	2.302976	down	1.0349579	down	2.1298664	down
7905929	1.5475854	down	6.4375386	up	2.7592466	up
7907349	1.6761777	up	1.6105281	up	2.1089993	up
7908347	4.075566	up	1.0819963	up	4.1298833	up
7909839	2.7696972	up	1.1232837	up	2.6691685	up
7912706	2.0000284	down	1.6192496	up	1.4111823	down
7914094	1.696644	up	1.1571207	up	2.0586839	up
7914603	1.5476654	up	2.0616717	up	2.191194	up
7915504	2.3806021	down	1.0994763	down	2.534039	down
7917503	1.3665904	up	2.4800422	up	3.297532	up
7917514	1.167309	up	2.9382727	up	2.6458046	up
7917516	1.598253	up	3.344233	up	3.9458985	up
7917530	1.3760818	up	3.8510222	up	5.3077455	up
7917532	1.1760712	down	2.625148	up	2.2186656	up
7918725	1.9282532	up	1.0030944	down	2.057421	up
7919589	2.6767964	down	1.0022985	up	2.5396922	down
7919612	2.9894493	down	1.039097	down	2.8355222	down
7919614	2.525579	down	1.0614165	up	2.400456	down
7919747	2.5292373	up	2.0141478	up	3.0402343	up
7919749	1.9127309	up	1.8552912	up	2.7877865	up
7920341	1.8830367	up	1.0794905	down	2.010113	up
7920401	2.2361987	down	1.0271832	up	2.2903645	down
7921621	2.1513727	up	1.1806052	up	2.6016772	up
7921916	2.0903435	up	1.1108546	up	1.8315898	up
7922229	1.0442609	up	2.8259149	up	1.6793075	up
7923753	1.0844691	up	1.9498838	up	2.0491138	up
7924029	2.2568316	down	2.1320176	up	1.1297443	down
7925531	1.9845777	up	1.0760733	up	2.0994337	up
7926875	1.2828289	up	3.7852602	up	3.3536305	up
7926900	1.0720813	up	2.2228436	up	2.4233856	up
7928308	3.459583	down	1.1017288	up	2.9193566	down
7928489	4.4900985	up	1.0820185	up	4.404106	up
7930074	1.1109186	down	2.3015468	up	1.4743282	up
7933043	2.5032167	up	1.0227156	up	2.3368337	up
7933228	1.917342	up	1.0587547	up	2.0620441	up
7933707	1.9599841	down	1.159282	down	2.203837	down
7938301	1.9903129	down	1.2492743	down	2.086512	down
7938396	1.124111	up	2.1464915	up	1.8625218	up
7938702	1.431981	up	1.7606944	up	2.232552	up
7939341	1.511618	up	1.4451082	up	2.0299945	up
7939374	3.2268572	up	1.3541443	up	3.5108619	up
7939434	1.8220451	up	1.1878307	up	2.0116203	up
7941104	2.0251718	down	1.0550069	down	2.0994446	down
7941136	2.0646636	down	1.038632	up	2.078328	down
7941621	2.5391202	down	1.0836924	up	2.4729493	down
7942168	2.4605563	down	1.1311376	down	2.6599412	down
7942596	2.6515155	down	1.2202382	down	2.8673735	down
7942824	2.386951	down	1.105973	down	2.3018968	down
7943367	3.324317	up	2.5973916	up	7.6375036	up
7943715	1.6618454	up	2.2585733	up	2.316999	up

7943890	2.6156194	up	1.4511335	up	2.4573867	up
7945283	2.028868	down	1.3203614	down	2.133306	down
7945521	2.1493819	down	1.069956	down	2.254094	down
7948565	2.2612264	down	1.0257081	down	2.525728	down
7949603	2.4510283	down	1.1628865	down	2.5776012	down
7950248	1.7940098	up	1.0211515	down	2.002605	up
7952986	3.0668278	up	1.5682039	up	4.2659307	up
7953508	2.2922707	down	1.1503038	down	2.0646195	down
7954029	1.6652582	down	1.3198634	down	2.321711	down
7954104	2.029076	up	1.0617579	up	1.9319618	up
7955589	1.0269986	up	2.7261426	up	1.7611876	up
7956908	2.0496678	up	2.2404115	up	4.430343	up
7957298	1.9047928	up	1.7775489	up	2.7833207	up
7958019	1.2645311	up	2.2547336	up	2.3101542	up
7959146	2.6179366	down	1.2201854	down	2.474122	down
7962579	1.538658	up	1.8048514	up	2.2970684	up
7966150	1.7506326	up	1.7266798	up	2.3066595	up
7966996	2.2771075	down	1.093994	down	2.2975233	down
7968484	2.3353126	up	1.1211064	up	2.2901547	up
7968883	1.7834044	up	1.8554488	up	2.2668862	up
7970507	2.0206625	up	1.0425197	up	1.7563987	up
7971620	1.9085242	up	1.0317934	down	2.037839	up
7972826	1.470991	down	2.217695	up	1.2803427	down
7974335	2.487498	up	1.6206765	up	3.1684723	up
7975121	2.199798	down	1.1052421	down	2.3142388	down
7975602	2.355822	down	1.0838075	down	2.5195658	down
7976451	1.7626507	up	2.389506	up	3.9482453	up
7976567	1.0485333	up	2.4624784	up	1.9107491	up
7976648	1.9178782	up	1.0598499	up	2.1269221	up
7977046	1.5271263	down	3.1619003	up	1.6030178	up
7977507	2.4097528	down	1.1299441	down	2.345211	down
7978644	1.0085242	up	2.804837	up	2.6916158	up
7979269	1.1420517	up	2.468517	up	2.1813076	up
7981111	1.8125315	up	1.1805418	up	2.097866	up
7982248	2.3360312	up	1.0115979	down	2.0646968	up
7982269	2.3082364	up	1.0486037	down	2.0020556	up
7982507	2.1315823	down	1.1567513	down	2.123176	down
7982753	1.7968625	down	1.1162198	down	2.0254605	down
7983828	2.005136	up	1.0144621	down	1.7416393	up
7986411	1.8784914	up	1.0884192	up	2.0557804	up
7987025	2.3072028	up	1.0488248	down	2.00032	up
7987135	2.6541104	up	1.1063641	up	2.1349857	up
7987572	2.2362099	down	1.1875457	down	2.689146	down
7987772	1.9356287	down	1.0899607	down	2.0403447	down
7989132	2.071413	up	1.0722613	down	1.8068324	up
7989661	1.9704598	down	1.0682566	down	2.1547027	down
7989849	1.6664084	up	1.5956157	up	2.2690988	up
7989883	1.3052235	up	2.176453	up	2.6824837	up
7992021	1.7426167	down	1.1341977	down	2.0554652	down
7994659	2.2853608	down	1.1347035	down	2.2948787	down
7996516	1.9245323	down	1.0466641	up	2.0166407	down
7998664	2.1429653	down	1.0067971	up	2.0501835	down
8000998	2.066618	down	1.1965871	down	2.026479	down
8001547	2.3900156	down	1.1624962	down	2.7758002	down
8002266	2.1820292	down	1.1211561	down	2.2714992	down
8003332	2.7644083	down	1.0412112	down	2.8171442	down
8003401	2.3409479	down	1.0219353	down	2.4409983	down
8004167	3.0913694	down	1.2374643	down	3.0261133	down
8004271	2.5635955	down	1.0959291	down	2.6843307	down
8004905	1.208083	down	2.1330562	up	1.4593081	up
8005166	2.0766997	down	1.0122373	down	2.290905	down
8005399	1.8979347	down	1.0922496	down	2.013154	down
8005471	2.4255216	down	1.093034	down	2.3599038	down
8005547	6.352794	down	1.09853	down	4.0271854	down
8005553	6.3538485	down	1.099	down	4.0294127	down
8005683	1.4666522	up	1.1979381	up	2.4819431	up
8005736	2.0540016	down	1.122374	down	1.9565161	down
8006690	1.9775531	down	1.0493935	down	2.060545	down
8007212	1.2380027	up	3.2527473	up	2.6420288	up
8008052	1.8143865	up	1.242577	up	2.074978	up
8008297	2.0400062	down	1.1738588	down	1.9259623	down
8009727	2.0826	down	1.0671111	down	2.2856803	down
8013035	2.1016817	up	1.070621	down	1.842002	up
8013323	6.3511243	down	1.0990334	down	4.02931	down

8013325	6.351667	down	1.098883	down	4.02888	down
8013329	6.3491282	down	1.0988333	down	4.027393	down
8014197	1.4563494	up	1.9991854	up	3.0008686	up
8017651	1.3719014	up	1.9937439	up	2.1026475	up
8018975	2.5436165	down	1.0248704	down	2.5447247	down
8020025	1.8253485	up	1.0100636	down	2.169608	up
8021727	2.1405797	down	1.0042708	down	2.2040265	down
8023401	1.8243015	up	1.114739	up	2.0873282	up
8024089	2.7726822	down	1.1032242	down	2.759471	down
8025395	2.4366035	down	1.0967386	down	2.3431013	down
8025601	1.1610497	down	3.4296024	up	2.6978254	up
8025612	1.1124451	down	3.111842	up	1.4781314	up
8027117	2.086925	down	1.0410175	down	2.0646763	down
8027368	2.1467955	up	1.1795384	down	1.8845503	up
8027884	2.6170576	down	1.1684384	down	3.403731	down
8027996	2.3961644	down	1.0146303	down	3.1090896	down
8028908	1.4524866	down	2.8098178	up	1.2585241	up
8030128	1.2058955	up	2.021642	up	1.6624088	up
8030171	1.9898623	down	1.04835	down	2.049528	down
8032718	2.1748054	down	1.0316616	up	1.87891	down
8032863	2.2743945	down	1.0570076	down	2.3511078	down
8032909	2.5588176	down	1.0892628	down	2.719951	down
8033257	1.3112069	up	2.3351917	up	2.6158745	up
8034021	2.1243675	down	1.0924027	down	2.05077	down
8034034	2.1524968	down	1.0921302	down	2.044694	down
8034416	1.9804751	down	1.032919	down	2.043322	down
8034631	1.9437693	down	1.0017105	down	2.0745587	down
8034920	2.6601653	down	1.037962	down	2.7285044	down
8035600	1.9375521	down	1.0327528	up	2.1491556	down
8036207	1.1327382	down	2.3366854	up	1.6327901	up
8036318	2.0876863	up	1.0142809	down	1.9493417	up
8037301	1.25914	down	2.496095	up	1.1884428	up
8041168	2.033217	up	1.2942848	up	2.143209	up
8041204	2.1416798	down	1.0080991	up	2.0482297	down
8042144	1.8483477	up	2.2133129	up	3.182102	up
8044333	1.8436674	up	1.1324173	up	2.0276656	up
8044450	2.3124166	up	1.0266119	up	2.2341766	up
8047606	1.9721545	up	1.1950548	up	2.033575	up
8048717	1.1523522	up	3.93398	up	3.1351802	up
8048864	2.008242	up	20.377653	up	18.77999	up
8049073	2.703198	down	1.1918703	down	2.828992	down
8051443	1.9234077	up	1.018277	up	2.0088701	up
8051583	2.4936876	up	1.999008	up	2.0392249	up
8052669	1.9956686	up	1.4312721	up	2.5892189	up
8053406	2.0131967	down	1.0350666	up	2.065407	down
8053741	2.5041878	up	1.0295722	down	2.205934	up
8054519	1.8440013	up	1.1300061	up	2.0267556	up
8055208	1.9858154	down	1.2216893	down	2.177338	down
8056285	1.510869	up	1.5096546	up	2.086879	up
8056977	1.7429456	up	1.382266	up	2.157843	up
8058201	2.3484	up	1.0676371	up	2.015425	up
8058390	1.7030606	up	1.9540936	up	3.3529017	up
8058477	1.1293082	up	2.8967788	up	2.5349073	up
8058914	2.3439815	down	1.063061	down	2.2140217	down
8059111	2.1503854	down	1.1258776	down	2.3653705	down
8060949	1.904496	up	1.0096996	up	2.0315578	up
8063345	1.9794463	down	1.2107245	up	2.1896925	down
8064388	1.1951004	down	2.267441	up	1.2797167	up
8064502	2.193757	down	1.0455247	up	2.0931487	down
8064978	1.1098151	up	2.1242259	up	1.6428797	up
8065596	2.387699	down	1.3158562	down	2.3994694	down
8065855	2.2653863	down	1.2135451	down	2.4632945	down
8066697	2.458562	down	1.2414794	up	2.1945846	down
8067040	1.9787192	up	1.1510756	up	2.3025212	up
8068238	1.5279917	up	2.0454378	up	2.3863633	up
8068460	1.9296855	up	1.1401004	up	2.0098007	up
8069057	2.6664457	down	1.088534	down	2.84374	down
8070194	2.0045133	up	1.2685838	up	2.0577865	up
8070720	1.0423127	down	2.06424	up	1.5587813	up
8071737	2.7539003	down	1.1042078	down	2.6744292	down
8074969	2.0567482	down	1.1674533	down	2.1489017	down
8076209	1.9536283	down	1.1084241	down	2.0020297	down
8077786	1.168828	down	2.5607796	up	1.5831003	up
8078300	1.729967	down	1.2361484	down	2.1334038	down

8080781	2.3361084	up	1.0402596	up	1.8495088	up
8081386	1.276441	up	3.7740483	up	3.5164177	up
8082350	2.0047944	down	1.037563	down	2.0723724	down
8083792	2.0868323	down	1.2191485	down	2.176073	down
8084878	2.3524845	up	1.121406	down	2.0166926	up
8087576	2.2458987	down	1.1597097	down	2.251189	down
8088128	1.7982833	up	1.1919836	up	2.1717465	up
8091723	1.1514354	up	2.1972134	up	1.871209	up
8093126	2.196205	up	1.0564064	up	2.059674	up
8094599	1.4787436	up	1.7595485	up	2.6896172	up
8095187	2.1469867	up	1.471999	up	2.9328854	up
8095680	1.7124621	up	4.275438	up	5.6811523	up
8095697	1.4072051	up	4.810818	up	5.812699	up
8095868	1.924822	up	2.3237202	up	3.76869	up
8097461	1.0874869	down	2.0903218	up	1.8540576	up
8097468	1.5473449	up	1.7545134	up	3.391624	up
8098690	1.0746492	up	2.689937	up	2.2798815	up
8098758	3.0247314	up	1.0620939	up	2.9699917	up
8099850	2.096833	up	1.7529854	up	2.5725706	up
8100003	2.6092296	up	1.3821673	up	2.6088207	up
8100977	1.4884754	up	1.8118126	up	2.4600766	up
8100994	1.47793	up	2.7908084	up	3.3017724	up
8102362	1.0985043	up	2.9115393	up	2.8650837	up
8103911	1.8260171	up	1.2546285	up	2.5477805	up
8104926	2.126884	up	1.1960171	down	1.2585322	up
8110022	2.0412261	down	1.0610658	down	2.0997744	down
8111812	1.9009086	up	1.1688567	up	2.1001616	up
8112274	1.2903603	up	2.500476	up	2.1461499	up
8112668	1.5968981	up	1.6458184	up	2.9795928	up
8113709	1.4863268	up	2.248343	up	2.3264732	up
8114006	2.2371616	up	1.0745023	down	1.9061667	up
8114010	1.2329897	up	6.85351	up	5.52027	up
8115831	2.1827073	down	1.0006156	down	2.1346173	down
8116227	1.553573	up	1.3229613	up	2.019612	up
8116910	1.8413328	up	2.373097	up	3.3137841	up
8116983	1.0514922	down	2.888179	up	2.3649936	up
8116992	1.0636276	up	1.9691668	up	2.1072226	up
8117594	2.3129609	up	1.1213775	down	2.4374418	up
8118007	2.4401696	down	1.1209488	down	2.4752946	down
8118345	1.1542864	down	2.388828	up	1.3842418	up
8118634	2.0405197	down	1.0145099	up	2.0696998	down
8119088	1.0806347	down	2.3368418	up	1.1447896	up
8121884	2.8794725	up	2.296197	up	5.080227	up
8122265	1.4765267	up	9.642801	up	8.653875	up
8123148	2.0290387	down	1.0798239	down	2.0732186	down
8123315	1.6602969	up	1.2701585	up	2.0312552	up
8124388	2.3168983	down	1.3381572	down	2.4078984	down
8124498	2.5198102	up	1.2123121	down	2.360135	up
8124531	2.6456537	down	1.4648201	down	3.4596224	down
8126066	2.4061582	down	1.0757681	down	2.4923935	down
8126658	2.9006386	down	1.1597369	down	2.7057288	down
8126666	1.4438196	down	2.2188869	up	1.1237485	up
8127502	2.0399754	up	1.2295152	up	2.4513066	up
8129953	2.2557268	up	3.160131	up	4.3018303	up
8130556	1.4069401	down	3.5534508	up	2.1185637	up
8131666	1.2694384	up	2.5857184	up	2.6026847	up
8131803	1.2556094	up	3.4094114	up	3.4316628	up
8133030	2.048913	up	1.0109047	down	2.1020977	up
8133345	2.3527908	down	1.0515833	down	2.3386137	down
8134339	1.9480876	up	1.1676682	up	2.0625093	up
8135967	2.0706067	up	1.2532215	up	1.9676554	up
8136983	2.266387	up	1.1545	up	2.3133092	up
8143629	2.2727401	up	1.1597842	up	2.440964	up
8145259	2.0203755	down	1.0772828	down	2.0203438	down
8145795	12.270263	up	1.2416594	down	10.261096	up
8146788	2.5862873	up	1.0239607	down	2.3743205	up
8147079	2.1299953	up	1.0613607	down	2.1301715	up
8147206	1.2451265	up	2.3153667	up	2.5529838	up
8148728	2.9833493	down	1.0713997	down	2.9632113	down
8151401	2.1500995	up	1.0319717	up	2.1340044	up
8151549	1.8844202	up	1.0681638	up	2.0102491	up
8151816	1.2920676	up	1.5235186	up	2.0517359	up
8154233	1.4356257	up	1.9324012	up	2.4501698	up
8155250	2.170658	down	1.118608	down	2.240621	down

8158684	2.564328	up	4.386171	up	9.893243	up
8159337	2.6434324	down	1.1418958	down	2.583625	down
8159609	1.9586276	down	1.1788433	down	2.0851092	down
8159642	2.287777	down	1.0769913	down	2.2771919	down
8160835	2.280975	down	1.1827819	down	2.4184878	down
8160953	2.014237	down	1.0072609	down	2.1262584	down
8161024	4.0678344	down	1.1375593	down	4.36047	down
8161526	1.1098498	down	3.330245	up	1.5501455	up
8163930	2.6951709	down	1.1293191	down	2.70201	down
8164428	2.5133436	down	1.1026566	down	2.5356393	down
8167790	2.1549838	down	1.1276081	down	2.1596563	down
8168691	2.0594351	up	1.0690289	up	2.1869152	up
8170882	2.1394508	down	1.0332601	down	2.0882452	down
8171024	2.723695	up	1.0095226	up	2.7475665	up
8172088	1.7000552	up	1.4742575	up	2.5048892	up
8172358	2.0651014	down	1.0647056	down	2.1897547	down
8172905	2.5166852	down	1.0118152	down	2.452382	down
8173673	2.367521	up	1.0148996	up	2.2483373	up
8174893	2.0969703	up	1.0632219	up	2.1444426	up
8176566	2.9333315	up	1.0705352	down	2.3578038	up
8177885	2.4394307	down	1.1227775	down	2.469719	down
8178115	1.141997	down	2.4205186	up	1.3821541	up
8178244	2.0859368	down	1.0207036	up	2.113231	down
8178676	2.0922308	down	1.0672346	down	2.2846334	down
8179205	2.438852	down	1.1211872	down	2.4728563	down
8179827	2.0064256	down	1.0132763	down	1.9728495	down
