# Supplementary material for: The Receptor Tyrosine Kinase FGFR4 Negatively Regulates NF-kappaB Signaling
Source: PLoS One. 2010 Dec 22;5(12):e14412. doi: 10.1371/journal.pone.0014412 (PMC3008709; doi:10.1371/journal.pone.0014412)
Supplement: Table S2 — Microarray Expression Data of DU145 Cells Using TNFalpha as Control. (0.12 MB PDF) [file pone.0014412.s002.pdf]

Supplemental Table 2. Microarray Expression Data of DU145 Cells Using TNFalpha as Control.

Samples: DU145 cells collected 1.5 h after treatments: **1)** Mock; **2)** FGF19; **3)** TNFalpha; **4)** FGF19 + TNFalpha. Duplicate samples collected; analyzed on 8 chips; grouped in 4 treatments for analysis.

Analysis: Analyzed in GeneSpringGX: 1148 out of 24,220 probesets satisfy corrected p-value cut-off of 0.015 using Oneway ANOVA Asymtotic p-value computation; of these, 266 satisfy fold-change cut-off of 2.0 using [TNFalpha] as the control condition. All genes that satisfy a fold-change cut-off of 2.0 in Sample 4 [FGF19 + TNFalpha] relative to Sample 3 [TNFalpha] as the control condition are presented here.

Transcript Cluster ID refers to Affymetrix GeneChip Human Gene 1.0 ST Array Human (cat# 901085). Information on probesets is available at http://media.affymetrix.com in the file HuGene-1_0-v1.na30.hg19.transcript.csv.zip

Gene Ontology information from http://www-bimas.cit.nih.gov/cards// . For genes with multiple Gene Ontology designations, a maximum of 5 are shown. Values for Fold Change in expression are rounded to two decimal places. All genes with a role in cell cycle, cell proliferation, apoptosis, NFkappaB cascade, cell death are highlighted and are discussed in more detail in Table 2.

Transcript Cluster Id	Fold change [FGF+TNF] vs [TNF])	Regulation [FGF+TNF] vs [TNF]	Protein	RefSeq	Gene Ontology (GO) designation	Biological or molecular process terms
8145795	12.74	UP	LOC100293539 // similar to ribosomal protein 10	XM_002346094	N/A	N/A
7908347	3.82	UP	OCLM // oculomedin	NM_022375	GO:0007601	visual perception
8124498	2.86	UP	ZNF204 // zinc finger protein 204 pseudogene	NR_002722	N/A	N/A
8098758	2.80	UP	ZNF721 // zinc finger protein 721	NM_133474	GO:0006350<br>GO:0006355	transcription<br>regulation of transcription, DNA-dependent
8117594	2.73	UP	HIST1H2BM // histone cluster 1, H2bm	NM_003521	GO:0006334	nucleosome assembly
7952986	2.72	UP	HSN2 // hereditary sensory neuropathy, type II	NM_213655	GO:0048666	neuron developmen
7899455	2.51	UP	PHACTR4 // phosphatase and actin regulator 4	NM_001048183	GO:0003779<br>GO:0004864	actin binding<br>phosphoprotein phosphatase inhibitor activity
8053741	2.27	UP	ANKRD20B // ankyrin repeat domain 20B	NR_003366	N/A	N/A
8147079	2.26	UP	LRRCC1 // leucine rich repeat and coiled-coil domain containing 1	NM_033402	GO:0007049<br>GO:0007067<br>GO:0051301	cell cycle<br>mitosis<br>cell division
8027368	2.22	UP	ZNF254 // zinc finger protein 254	NM_203282	GO:0000122<br>GO:0006350<br>GO:0006355	negative regulation of transcription from RNA polymerase II promoter<br>transcription<br>regulation of transcription, DNA-dependent
8173673	2.22	UP	ATRX // alpha thalassemia/mental retardation syndrome X-linked (RAD54 homolog, S. cerevisiae)	NM_000489	GO:0006281<br>GO:0006306<br>GO:0006310<br>GO:0006355<br>GO:0006974	DNA repair<br>DNA methylation<br>DNA recombination<br>regulation of transcription, DNA-dependent<br>response to DNA damage stimulus
8044450	2.18	UP	ZC3H6 // zinc finger CCCH-type containing 6	NM_198581	GO:0003676<br>GO:0008270<br>GO:0046872	nucleic acid binding<br>zinc ion binding<br>metal ion binding
7920341	2.17	UP	GATAD2B // GATA zinc finger domain containing 2B	NM_020699	GO:0006350<br>GO:0006355	transcription<br>regulation of transcription, DNA-dependent
8013305	2.13	UP	ZNF286A // zinc finger protein 286A	NM_020652	GO:0006350<br>GO:0006355	transcription<br>regulation of transcription, DNA-dependent
8105523	2.13	UP	KIF2A // kinesin heavy chain member 2A	NM_004520	GO:0007018<br>GO:0007275<br>GO:0007399<br>GO:0030154	microtubule-based movement<br>multicellular organismal development<br>nervous system development<br>cell differentiation

Transcript Cluster Id	Fold change [FGF+TNF] vs [TNF])	Regulation [FGF+TNF] vs [TNF]	Protein	RefSeq	Gene Ontology (GO) designation	Biological or molecular process terms
8133030	2.13	UP	GABPA // GA binding protein transcription factor, alpha subunit 60kDa	NM_002040	GO:0000122	negative regulation of transcription from RNA polymerase II promoter
					GO:0001701	in utero embryonic development
					GO:0006350	transcription
8143629	2.10	UP	OR2A9P // olfactory receptor, family 2, subfamily A9 pseudogene	NR_002157	N/A	N/A
7971620	2.10	UP	KPNA3 // karyopherin alpha 3 (importin alpha 4)	NM_002267	GO:0006461	protein complex assembly
					GO:0006607	NLS-bearing substrate import into nucleus
					GO:0006886	intracellular protein transport
8151401	2.07	UP	UBE2W // ubiquitin-conjugating enzyme E2W (putative)	NM_001001481	GO:0019941	modification-dependent protein catabolic process
					GO:0043687	post-translational protein modification
					GO:0051246	regulation of protein metabolic process
8097586	2.07	UP	GAB1 // GRB2-associated binding protein 1	NM_207123	GO:0007173	epidermal growth factor receptor signaling pathway
					GO:0008283	cell proliferation
					GO:0008286	insulin receptor signaling pathway
7918725	2.06	UP	TRIM33 // tripartite motif-containing 33	NM_015906	GO:0006355	regulation of transcription, DNA-dependent
					GO:0016481	negative regulation of transcription
					GO:0019941	modification-dependent protein catabolic process
8168691	2.05	UP	DIAPH2 // diaphanous homolog 2 (Drosophila)	NM_006729	GO:0000910	cytokinesis
					GO:0007275	multicellular organismal development
					GO:0016043	cellular component organization
					GO:0030036	actin cytoskeleton organization
					GO:0030154	cell differentiation
7950248	2.04	UP	FCHSD2 // FCH and double SH3 domains 2	NM_014824	GO:0005515	protein binding
7968484	2.04	UP	BRCA2 // breast cancer 2, early onset	NM_000059	GO:0000724	double-strand break repair via homologous recombination
					GO:0001556	oocyte maturation
					GO:0001833	inner cell mass cell proliferation
					GO:0006289	nucleotide-excision repair
					GO:0006310	DNA recombination
7993664	2.04	UP	CP110 // CP110 protein	NM_014711	GO:0032465	regulation of cytokinesis
					GO:0051298	centrosome duplication
8103911	2.03	UP	IRF2 // interferon regulatory factor 2	NM_002199	GO:0000122	negative regulation of transcription from RNA polymerase II promoter
					GO:0006350	transcription
					GO:0006355	regulation of transcription, DNA-dependent
					GO:0008283	cell proliferation
8014115	2.03	UP	MYO1D // myosin ID	NM_015194	GO:0000166	nucleotide binding
					GO:0003774	motor activity
					GO:0003779	actin binding
					GO:0005516	calmodulin binding
					GO:0005524	ATP binding
8056060	2.02	UP	BAZ2B // bromodomain adjacent to zinc finger domain, 2B	NM_013450	GO:0006338	chromatin remodeling
					GO:0006350	transcription
					GO:0006355	regulation of transcription, DNA-dependent
8174893	2.02	UP	THOC2 // THO complex 2	NM_001081550	GO:0006338	chromatin remodeling
					GO:0006397	mRNA processing
					GO:0006810	transport
					GO:0008380	RNA splicing
					GO:0051028	mRNA transport
8060949	2.01	UP	ANKRD5 // ankyrin repeat domain 5	NM_022096	GO:0005509	calcium ion binding
7917433	2.01	UP	ODF2L // outer dense fiber of sperm tails 2-like	NM_020729	N/A	N/A
7976648	2.01	UP	CCNK // cyclin K	NM_001099402	GO:0000079	regulation of cyclin-dependent protein kinase activity
					GO:0006350	transcription
					GO:0006355	regulation of transcription, DNA-dependent
					GO:0006366	transcription from RNA polymerase II promoter
					GO:0007049	cell cycle
8136983	2.00	UP	OR2A20P // olfactory receptor, family 2, subfamily A20 pseudogene	NR_002158	N/A	N/A

Transcript Cluster Id	Fold change [FGF+TNF] vs [TNF])	Regulation [FGF+TNF] vs [TNF]	Protein	RefSeq	Gene Ontology (GO) designation	Biological or molecular process terms
8067040	2.00	UP	NFATC2 // nuclear factor of activated T-cells, cytoplasmic, calcineurin-dependent 2	NM_012340	GO:0001816	cytokine production
					GO:0006355	regulation of transcription, DNA-dependent
					GO:0045941	positive regulation of transcription
					GO:0045944	positive regulation of transcription from RNA polymerase II promoter
8161024	3.83	DOWN	RMRP // RNA component of mitochondrial RNA processing endoribonuclease	NR_003051	N/A	N/A
8005553	3.67	DOWN	SNORD3A // small nucleolar RNA, C/D box 3A	NR_006880	N/A	N/A
7928308	3.22	DOWN	DDIT4 // DNA-damage-inducible transcript 4	NM_019058	GO:0006915	apoptosis
					GO:0009968	negative regulation of signal transduction
8027996	3.06	DOWN	PSENEN // presenilin enhancer 2 homolog	NM_172341	GO:0006509	membrane protein ectodomain proteolysis
					GO:0007220	Notch receptor processing
					GO:0016485	protein processing
					GO:0042987	amyloid precursor protein catabolic process
					GO:0043085	positive regulation of catalytic activity
7972826	2.84	DOWN	C13orf29 // chromosome 13 open reading frame 29	NR_027701	N/A	N/A
8148728	2.77	DOWN	CYC1 // cytochrome c-1	NM_001916	GO:0006810	transport
					GO:0022900	electron transport chain
7919612	2.73	DOWN	HIST2H3D // histone cluster 2, H3d	NM_001123375	N/A	N/A
8066697	2.72	DOWN	SLC35C2 // solute carrier family 35, member C2	NM_173179	GO:0006810	transport
8003332	2.71	DOWN	MVD // mevalonate (diphospho) decarboxylase	NM_002461	GO:0006695	cholesterol biosynthetic process
					GO:0008299	isoprenoid biosynthetic process
					GO:0016310	phosphorylation
7941621	2.68	DOWN	DPP3 // dipeptidyl-peptidase 3	NM_005700	GO:0006508	proteolysis
8063345	2.65	DOWN	SNORD12C // small nucleolar RNA, C/D box 12C	NR_002433	N/A	N/A
8034920	2.63	DOWN	ILVBL // ilvB (bacterial acetolactate synthase)-like	NM_006844	GO:0008150	biological_process
8069057	2.61	DOWN	PFKL // phosphofructokinase, liver	NR_024108	GO:0005975	carbohydrate metabolic process
					GO:0006002	fructose 6-phosphate metabolic process
					GO:0006096	glycolysis
					GO:0009749	response to glucose stimulus
					GO:0046676	negative regulation of insulin secretion
					GO:0005975	carbohydrate metabolic process
7905085	2.55	DOWN	LOC388692 // hypothetical LOC388692	NR_027002	N/A	N/A
7919614	2.55	DOWN	HIST2H3A // histone cluster 2, H3a	NM_001005464	N/A	N/A
7919589	2.55	DOWN	HIST2H3D // histone cluster 2, H3d	NM_001123375	GO:0006334	nucleosome assembly
8024089	2.50	DOWN	WDR18 // WD repeat domain 18	NM_024100	N/A	N/A
8032909	2.50	DOWN	M6PRBP1 // perilipin 3	NM_005817	GO:0016192	vesicle-mediated transport
8018975	2.48	DOWN	LGALS3BP // lectin, galactoside-binding, soluble, 3 binding protein	NM_005567	GO:0006968	cellular defense response
					GO:0007155	cell adhesion
					GO:0007165	signal transduction
7948565	2.46	DOWN	CYBASC3 // cytochrome b, ascorbate dependent 3	NM_001161454	GO:0006810	transport
					GO:0022900	electron transport chain
8004271	2.45	DOWN	ACADVL // acyl-Coenzyme A dehydrogenase, very long chain	NM_000018	GO:0006629	lipid metabolic process
					GO:0006631	fatty acid metabolic process
					GO:0006635	fatty acid beta-oxidation
					GO:0015980	energy derivation by oxidation of organic compounds
					GO:0055114	oxidation reduction
7901110	2.44	DOWN	AKR1A1 // aldo-keto reductase family 1, member A1 (aldehyde reductase)	NM_006066	GO:0006006	glucose metabolic process
					GO:0019853	L-ascorbic acid biosynthetic process
					GO:0042840	D-glucuronate catabolic process
					GO:0046185	aldehyde catabolic process
					GO:0055114	oxidation reduction
8172905	2.42	DOWN	HSD17B10 // hydroxysteroid (17-beta) dehydrogenase 10	NM_004493	GO:0006629	lipid metabolic process
					GO:0008033	tRNA processing
					GO:0055114	oxidation reduction

Transcript Cluster Id	Fold change [FGF+TNF] vs [TNF])	Regulation [FGF+TNF] vs [TNF]	Protein	RefSeq	Gene Ontology (GO) designation	Biological or molecular process terms
8071737	2.42	DOWN	MIF // macrophage migration inhibitory factor (glycosylation-inhibiting factor)	NM_002415	GO:0001516<br>GO:0006954<br>GO:0007166<br>GO:0008283<br>GO:0043030	prostaglandin biosynthetic process<br>inflammatory response<br>cell surface receptor linked signal transduction<br>cell proliferation<br>regulation of macrophage activation
7924029	2.41	DOWN	LAMB3 // laminin, beta 3	NM_001017402	GO:0007155<br>GO:0008544	cell adhesion<br>epidermis development
8163930	2.39	DOWN	NDUFA8 // NADH dehydrogenase (ubiquinone) 1 alpha subcomplex, 8, 19kDa	NM_014222	GO:0006120<br>GO:0006810<br>GO:0022900	mitochondrial electron transport, NADH to ubiquinone<br>transport<br>electron transport chain
8003401	2.39	DOWN	APRT // adenine phosphoribosyltransferase	NM_000485	GO:0006166<br>GO:0006168<br>GO:0007625<br>GO:0009116	purine ribonucleoside salvage<br>adenine salvage<br>grooming behavior<br>nucleoside metabolic process
8001547	2.39	DOWN	PLLP // plasma membrane proteolipid (plasmolipin)	NM_015993	GO:0006811	ion transport
8149733	2.37	DOWN	TNFRSF10B // tumor necrosis factor receptor superfamily, member 10b	NM_003842	GO:0006919<br>GO:0007166<br>GO:0007250<br>GO:0008625<br>GO:0008633	activation of caspase activity<br>cell surface receptor linked signal transduction<br>activation of NF-kappaB-inducing kinase activity<br>induction of apoptosis via death domain receptors<br>activation of pro-apoptotic gene products
8124531	2.36	DOWN	HIST1H3I // histone cluster 1, H3i	NM_003533	N/A	N/A
7920401	2.35	DOWN	SLC39A1 // solute carrier family 39 (zinc transporter), member 1	NM_014437	GO:0006811<br>GO:0006829<br>GO:0015674<br>GO:0030001	ion transport<br>zinc ion transport<br>di-, tri-valent inorganic cation transport<br>metal ion transport
7942168	2.35	DOWN	FADD // Fas (TNFRSF6)-associated via death domain	NM_003824	GO:0007165<br>GO:0008625<br>GO:0008633<br>GO:0042981<br>GO:0043123	signal transduction<br>induction of apoptosis via death domain receptors<br>activation of pro-apoptotic gene products<br>regulation of apoptosis<br>positive regulation of I-kappaB kinase/NF-kappaB cascade
7942596	2.35	DOWN	SERPINH1 // serpin peptidase inhibitor, clade H (heat shock protein 47), member 1, (collagen binding protein 1)	NM_001235	GO:0006986<br>GO:0030199<br>GO:0032964<br>GO:0051604	response to unfolded protein<br>collagen fibril organization<br>collagen biosynthetic process<br>protein maturation
7905929	2.33	DOWN	EFNA1 // ephrin-A1	NM_004428	GO:0007267	cell-cell signaling
8126658	2.33	DOWN	SLC35B2 // solute carrier family 35, member B2	NM_178148	GO:0006810<br>GO:0043123<br>GO:0046963	transport<br>positive regulation of I-kappaB kinase/NF-kappaB cascade<br>3'-phosphoadenosine 5'-phosphosulfate transport
7975602	2.32	DOWN	ACOT2 // acyl-CoA thioesterase 2	NM_006821	GO:0000038<br>GO:0001676<br>GO:0006629<br>GO:0006637	very-long-chain fatty acid metabolic process<br>long-chain fatty acid metabolic process<br>lipid metabolic process<br>acyl-CoA metabolic process
8126066	2.32	DOWN	MTCH1 // mitochondrial carrier homolog 1	NM_014341	GO:0006810<br>GO:0006919<br>GO:0009966<br>GO:0043065<br>GO:0045161	transport<br>activation of caspase activity<br>regulation of signal transduction<br>positive regulation of apoptosis<br>neuronal ion channel clustering
7915504	2.30	DOWN	ELOVL1 // elongation of very long chain fatty acids	NM_022821	GO:0006633	fatty acid biosynthetic process
8164428	2.30	DOWN	TRUB2 // TruB pseudouridine (psi) synthase homolog 2 (E. coli)	NM_015679	GO:0001522<br>GO:0008033	pseudouridine synthesis<br>tRNA processing
7912706	2.29	DOWN	EPHA2 // EPH receptor A2	NM_004431	GO:0006468<br>GO:0007165<br>GO:0007275<br>GO:0030182<br>GO:0048013	protein amino acid phosphorylation<br>signal transduction<br>multicellular organismal development<br>neuron differentiation<br>ephrin receptor signaling pathway

Transcript Cluster Id	Fold change [FGF+TNF] vs [TNF])	Regulation [FGF+TNF] vs [TNF]	Protein	RefSeq	Gene Ontology (GO) designation	Biological or molecular process terms
8005166	2.26	DOWN	UBB // ubiquitin B	NM_018955	GO:0006464	protein modification process
					GO:0007049	cell cycle
					GO:0007411	axon guidance
					GO:0016567	protein ubiquitination
					GO:0030433	ER-associated protein catabolic process
8159337	2.26	DOWN	PMPCA // peptidase (mitochondrial processing) alpha	NM_015160	GO:0006508	proteolysis
7899016	2.26	DOWN	SDHD // succinate dehydrogenase complex, subunit D, integral membrane protein	NM_003002	GO:0006099	tricarboxylic acid cycle
					GO:0006810	transport
					GO:0022900	electron transport chain
8110755	2.24	DOWN	SLC12A7 // solute carrier family 12 (potassium/chloride transporters), member 7	NM_006598	GO:0006811	ion transport
					GO:0006813	potassium ion transport
					GO:0006814	sodium ion transport
					GO:0006821	chloride transport
8028908	2.23	DOWN	ITPKC // inositol 1,4,5-trisphosphate 3-kinase C	NM_025194	GO:0000166	nucleotide binding
					GO:0005516	calmodulin binding
					GO:0005524	ATP binding
					GO:0008440	inositol trisphosphate 3-kinase activity
					GO:0016301	kinase activity
8032863	2.22	DOWN	C19orf10 // chromosome 19 open reading frame 10	NM_019107	GO:0008284	N/A
8133345	2.22	DOWN	WBSCR22 // Williams Beuren syndrome chromosome region 22	NM_017528	GO:0008152	metabolic process
8035600	2.22	DOWN	TMEM161A // transmembrane protein 161A	NM_017814	N/A	N/A
7949603	2.22	DOWN	BRMS1 // breast cancer metastasis suppressor 1	NM_015399	GO:0008150	biological_process
					GO:0045786	negative regulation of cell cycle
8177885	2.20	DOWN	GTF2H4 // general transcription factor IIH, polypeptide 4, 52kDa	NM_001517	GO:0000718	nucleotide-excision repair, DNA damage removal
					GO:0006281	DNA repair
					GO:0006350	transcription
					GO:0006355	regulation of transcription, DNA-dependent
					GO:0006367	transcription initiation from RNA polymerase II promoter
8021727	2.19	DOWN	CNDP2 // CNDP dipeptidase 2 (metallopeptidase M20 family)	NM_018235	GO:0006508	proteolysis
8064502	2.19	DOWN	SNRPB // small nuclear ribonucleoprotein polypeptides B and B1	NM_003091	GO:0000387	spliceosomal snRNP biogenesis
					GO:0008380	RNA splicing
7900922	2.17	DOWN	ATP6V0B // ATPase, H+ transporting, lysosomal 21kDa, V0 subunit b	NM_004047	GO:0006811	ion transport
					GO:0015986	ATP synthesis coupled proton transport
					GO:0015992	proton transport
8005471	2.16	DOWN	RPS28 // ribosomal protein S28	NM_001031	GO:0006364	rRNA processing
					GO:0006414	translational elongation
					GO:0042274	ribosomal small subunit biogenesis
7941136	2.16	DOWN	C11orf2 // chromosome 11 open reading frame 2	NM_013265	GO:0006869	lipid transport
					GO:0008150	biological_process
					GO:0015031	protein transport
7965423	2.16	DOWN	BTG1 // B-cell translocation gene 1, anti-proliferative	NM_001731	GO:0006479	protein amino acid methylation
					GO:0008285	negative regulation of cell proliferation
					GO:0016477	cell migration
					GO:0030308	negative regulation of cell growth
					GO:0042981	regulation of apoptosis
8178244	2.16	DOWN	RING1 // ring finger protein 1	NM_002931	GO:0006350	transcription
					GO:0006355	regulation of transcription, DNA-dependent
					GO:0009952	anterior/posterior pattern formation
					GO:0016568	chromatin modification
					GO:0016574	histone ubiquitination
8161526	2.15	DOWN	LOC440896 // hypothetical LOC440896	AK127288	N/A	N/A
8009727	2.14	DOWN	ICT1 // immature colon carcinoma transcript 1	NM_001545	GO:0006415	translational termination
					GO:0008150	biological_process
8178676	2.14	DOWN	NEU1 // sialidase 1 (lysosomal sialidase)	NM_000434	GO:0008152	metabolic process
8053406	2.14	DOWN	RETSAT // retinol saturase (all-trans-retinol 13,14-reductase)	NM_017750	GO:0042572	retinol metabolic process
					GO:0055114	oxidation reduction

Transcript Cluster Id	Fold change [FGF+TNF] vs [TNF])	Regulation [FGF+TNF] vs [TNF]	Protein	RefSeq	Gene Ontology (GO) designation	Biological or molecular process terms
8025395	2.14	DOWN	RPS28 // ribosomal protein S28	NM_001031	GO:0006364	rRNA processing
					GO:0006414	translational elongation
					GO:0042274	ribosomal small subunit biogenesis
8115831	2.13	DOWN	DUSP1 // dual specificity phosphatase 1	NM_004417	GO:0006470	protein amino acid dephosphorylation
					GO:0006979	response to oxidative stress
					GO:0007049	cell cycle
					GO:0007242	intracellular signaling cascade
8159642	2.11	DOWN	TUBB2C // tubulin, beta 2C	NM_006088	GO:0006928	cell motion
					GO:0007018	microtubule-based movement
					GO:0042267	natural killer cell mediated cytotoxicity
					GO:0051258	protein polymerization
8160953	2.11	DOWN	PIGO // phosphatidylinositol glycan anchor biosynthesis, class O	NM_032634	GO:0008152	metabolic process
					GO:0016254	preassembly of GPI anchor in ER membrane
7996516	2.11	DOWN	PLEKHG4 // pleckstrin homology domain containing, family G (with RhoGef domain) member 4	NM_015432	GO:0008219	cell death
					GO:0035023	regulation of Rho protein signal transduction
7945521	2.11	DOWN	PDDC1 // Parkinson disease 7 domain containing 1	NM_182612	N/A	N/A
8025612	2.11	DOWN	ICAM4 // intercellular adhesion molecule 4	NM_022377	GO:0016337	cell-cell adhesion
8059111	2.10	DOWN	ABCB6 // ATP-binding cassette, sub-family B (MDR/TAP), member 6	NM_005689	GO:0006810	transport
					GO:0006879	cellular iron ion homeostasis
8037301	2.10	DOWN	LYPD3 // LY6/PLAUR domain containing 3	NM_014400	GO:0006928	cell motion
					GO:0007160	cell-matrix adhesion
7966996	2.10	DOWN	RPLP0 // ribosomal protein, large, P0	NM_053275	GO:0006414	translational elongation
					GO:0042254	ribosome biogenesis
					GO:0044419	interspecies interaction between organisms
7975121	2.09	DOWN	FNTB // farnesyltransferase, CAAX box, beta	NM_002028	GO:0008285	negative regulation of cell proliferation
					GO:0018347	protein amino acid farnesylation
					GO:0042060	wound healing
					GO:0048146	positive regulation of fibroblast proliferation
8036938	2.09	DOWN	ADCK4 // aarF domain containing kinase 4	NM_024876	GO:0004674	protein serine/threonine kinase activity
					GO:0016301	kinase activity
					GO:0016740	transferase activity
8058914	2.08	DOWN	AAMP // angio-associated, migratory cell protein	NM_001087	GO:0001525	angiogenesis
					GO:0007275	multicellular organismal development
					GO:0010595	positive regulation of endothelial cell migration
					GO:0014909	smooth muscle cell migration
					GO:0030154	cell differentiation
7942824	2.08	DOWN	RPS28 // ribosomal protein S28	NM_001031	GO:0006364	rRNA processing
					GO:0006414	translational elongation
					GO:0042274	ribosomal small subunit biogenesis
7977507	2.08	DOWN	RPPH1 // ribonuclease P RNA component H1	NR_002312	N/A	N/A
8034631	2.07	DOWN	STX10 // syntaxin 10	NM_003765	GO:0006886	intracellular protein transport
					GO:0048193	Golgi vesicle transport
8002370	2.07	DOWN	ST3GAL2 // ST3 beta-galactoside alpha-2,3-sialyltransferase 2	NM_006927	GO:0006040	amino sugar metabolic process
					GO:0006486	protein amino acid glycosylation
8041204	2.06	DOWN	SNORA10 // small nucleolar RNA, H/ACA box 10	NR_002327	N/A	N/A
7905881	2.06	DOWN	ADAM15 // ADAM metallopeptidase domain 15	NM_207196	GO:0006508	proteolysis
					GO:0007160	cell-matrix adhesion
8099029	2.06	DOWN	TNIP2 // TNFAIP3 interacting protein 2	NM_024309	GO:0007249	I-kappaB kinase/NF-kappaB cascade
8172358	2.06	DOWN	UXT // ubiquitously-expressed transcript	NM_153477	GO:0000226	microtubule cytoskeleton organization
					GO:0006457	protein folding
					GO:0047497	mitochondrion transport along microtubule
					GO:0051297	centrosome organization
8155096	2.06	DOWN	CREB3 // cAMP responsive element binding protein 3	NM_006368	GO:0006355	regulation of transcription, DNA-dependent
					GO:0006935	chemotaxis
					GO:0044419	interspecies interaction between organisms

Transcript Cluster Id	Fold change [FGF+TNF] vs [TNF])	Regulation [FGF+TNF] vs [TNF]	Protein	RefSeq	Gene Ontology (GO) designation	Biological or molecular process terms
8160835	2.04	DOWN	C9orf23 // chromosome 9 open reading frame 23	NM_148179	GO:0003676	nucleic acid binding
8119088	2.04	DOWN	CDKN1A // cyclin-dependent kinase inhibitor 1A (p21, Cip1)	NM_078467	GO:0000082	G1/S transition of mitotic cell cycle
					GO:0000086	G2/M transition of mitotic cell cycle
					GO:0006974	response to DNA damage stimulus
					GO:0007050	cell cycle arrest
					GO:0008285	negative regulation of cell proliferation
7901175	2.04	DOWN	TSPAN1 // tetraspanin 1	NM_005727	N/A	N/A
8065855	2.03	DOWN	EDEM2 // ER degradation enhancer, mannosidase alpha-like 2	NR_026728	GO:0004571	mannosyl-oligosaccharide 1,2-alpha-mannosidase activity
					GO:0005509	calcium ion binding
7959146	2.03	DOWN	NME2P1 // non-metastatic cells 2, protein (NM23B) expressed in, pseudogene 1	NR_001577	N/A	N/A
8019149	2.03	DOWN	SLC38A10 // solute carrier family 38, member 10	NM_001037984	GO:0006811	ion transport
					GO:0006814	sodium ion transport
					GO:0006865	amino acid transport
8002266	2.03	DOWN	CHTF8 // CTF8, chromosome transmission fidelity factor 8 homolog (S. cerevisiae)	NM_001039690	GO:0006260	DNA replication
					GO:0007049	cell cycle
8003467	2.02	DOWN	CHMP1A // chromatin modifying protein 1A	NM_001083314	GO:0006350	transcription
					GO:0007049	cell cycle
					GO:0007076	mitotic chromosome condensation
					GO:0015031	protein transport
					GO:0016192	vesicle-mediated transport
7994659	2.02	DOWN	MVP // major vault protein	NM_017458	GO:0015031	protein transport
					GO:0042493	response to drug
					GO:0051028	mRNA transport
					GO:0065002	intracellular protein transmembrane transport
8170882	2.02	DOWN	ATP6AP1 // ATPase, H+ transporting, lysosomal accessory protein 1	NM_001183	GO:0006811	ion transport
					GO:0015986	ATP synthesis coupled proton transport
					GO:0015992	proton transport
7989661	2.02	DOWN	OAZ2 // ornithine decarboxylase antizyme 2	NM_002537	GO:0006595	polyamine metabolic process
8156290	2.00	DOWN	CKS2 // CDC28 protein kinase regulatory subunit 2	NM_001827	GO:0000079	regulation of cyclin-dependent protein kinase activity
					GO:0007049	cell cycle
					GO:0007051	spindle organization
					GO:0007127	meiosis I
					GO:0008283	cell proliferation
8155250	2.00	DOWN	GRHPR // glyoxylate reductase/hydroxypyruvate reductase	NM_012203	GO:0007588	excretion
					GO:0055114	oxidation reduction
7992414	2.00	DOWN	TBL3 // transducin (beta)-like 3	NM_006453	GO:0006364	rRNA processing
					GO:0007199	G-protein signaling, coupled to cGMP nucleotide second messenger
